# Group recommender systems for tourism: how does personality predict preferences for attractions, travel motivations, preferences and concerns?

**DOI:** 10.1007/s11257-023-09361-2

**Published:** 2023-05-15

**Authors:** Patrícia Alves, Helena Martins, Pedro Saraiva, João Carneiro, Paulo Novais, Goreti Marreiros

**Affiliations:** 1 ALGORITMI Research Centre/LASI, University of Minho, Guimarães, Portugal; 2 GECAD/LASI, ISEP, Polytechnic of Porto, Porto, Portugal; 3 CEOS.PP, ISCAP, Polytechnic of Porto, Porto, Portugal; 4 Faculty of Psychology and Education Sciences of the University of Porto, Porto, Portugal

**Keywords:** Group recommender systems, Personality, Tourist preferences, Travel motivations, Travel concerns, Affective computing

## Abstract

To travel in leisure is an emotional experience, and therefore, the more the information about the tourist is known, the more the personalized recommendations of places and attractions can be made. But if to provide recommendations to a tourist is complex, to provide them to a group is even more. The emergence of personality computing and personality-aware recommender systems (RS) brought a new solution for the cold-start problem inherent to the conventional RS and can be the leverage needed to solve conflicting preferences in heterogenous groups and to make more precise and personalized recommendations to tourists, as it has been evidenced that personality is strongly related to preferences in many domains, including tourism. Although many studies on psychology of tourism can be found, not many predict the tourists’ preferences based on the Big Five personality dimensions. This work aims to find how personality relates to the choice of a wide range of tourist attractions, traveling motivations, and travel-related preferences and concerns, hoping to provide a solid base for researchers in the tourism RS area to automatically model tourists in the system without the need for tedious configurations, and solve the cold-start problem and conflicting preferences. By performing Exploratory and Confirmatory Factor Analysis on the data gathered from an online questionnaire, sent to Portuguese individuals from different areas of formation and age groups (*n* = 1035), we show all five personality dimensions can help predict the choice of tourist attractions and travel-related preferences and concerns, and that only neuroticism and openness predict traveling motivations.

## Introduction

The last two decades have shown that personalization is the key to deliver the best recommendations in Recommender Systems (RS) (Chen et al. 2016; Poon and Huang 2017; Tkalcic and Chen 2015; Tkalcic et al. 2016; Tondello et al. 2017). Therefore, the more about the user is known the more accurate and tailored recommendations can be made. But if to provide tailored individual recommendations is complex, to provide them to groups is even more (Masthoff 2015). The tourism industry has many variables making it a very complex topic, which is aggravated when groups of tourists, that need a travel plan and to get accompanied in their excursions, are involved, particularly due to the group’s heterogeneity and conflicting preferences (Nguyen and Ricci 2018), making Group Recommender Systems (GRS) an important and challenging area of RS (Abolghasemi et al. 2022a, b; Castro et al. 2015; Delic and Masthoff 2018; Masthoff 2011; McCarthy et al. 2006).

The advances in mobile technologies, like smartphones and wearable devices, make it possible to collect users’ detailed data (Adomavicius and Tuzhilin 2011), such as contextual information, identity, preferences and interactions with the mobile device, which in turn can be used to provide and improve recommendations. Also, the emergence and increasing use of social media (e.g., social networks, virtual game worlds, content communities) make it easy to obtain more personal information (Kaplan and Haenlein 2010). Interestingly, these advances are coincident with the sudden increase in interest for personality computing in the early 2000s (Vinciarelli and Mohammadi 2014), and consequently to the recent proliferation of personality-aware RS (Dhelim et al. 2021), which can easily be explained by its own definition: “personality is the sum total of the actual or potential behavior patterns of the organism, as determined by heredity and environment” (Eysenck 1998), meaning each individual has her own behavior patterns, which are considered relatively stable over time across different situations (McCrae and Costa Jr 1997), i.e., “an individual’s behavior naturally varies somewhat from occasion to occasion, but… there is a core of consistency which defines the individual’s ‘true nature’” (Matthews et al. 2003). These behavior patterns were summarized into five universal personality dimensions by Costa and MacCrae (1992): Openness to Experience, Conscientiousness, Extraversion, Agreeableness and Neuroticism, being the Five-Factor Model (FFM), or Big Five, recognized as the most widely accepted model to represent them (Dhelim et al. 2021; Digman 1990; Matz et al. 2016), and therefore were adopted in this study. Each factor is defined by six traits/facets (Costa and MacCrae 1992), resulting in a total of 30 traits, which are more granular, better characterizing a person (see Table 1).

**Table 1:** Personality dimensions and their respective six traits (adapted from Costa and MacCrae (1992))

Neuroticism	Extraversion	Openness to experience	Agreeableness	Conscientiousness
Anxiety	Friendliness	Imagination	Trust	Self-efficacy
Anger	Gregariousness	Artistic interests	Morality	Orderliness
Depression	Assertiveness	Emotionality	Altruism	Dutifulness
Self-consciousness	Activity level	Adventurousness	Cooperation	Achievement-striving
Immoderation	Excitement-seeking	Intellect	Modesty	Self-discipline
Vulnerability	Cheerfulness	Liberalism	Sympathy	Cautiousness

Personality can be useful in different areas of RS (Ning et al. 2019; Tkalcic and Chen 2015) to help overcome challenges related to user modeling (Dhelim et al. 2021), since it is strongly related to the users’ preferences (Cantador et al. 2013; Martijn et al. 2022), i.e., users with similar personalities tend to choose similar items or contents (Cantador et al. 2013). For example, extraverts who are dependent on warmth and gregariousness tend to enjoy popular music, and persons who score high on excitement-seeking tend to prefer rock music (Cantador et al. 2013; Rawlings and Ciancarelli 1997). In games, extraverts are more inclined to group activities than solo activities (Yee et al. 2011). Even certain features of Instagram® pictures are related to personality traits (Ferwerda et al. 2015). Being personality an enduring characteristic of humans (Costa and McCrae 1988), it should be a better predictor of the tourist behavior than demographics like age, income, etc., as they are more liable to change over time (Jani 2014b).

Although personality is still a growing topic in RS (Dhelim et al. 2021; Jackson and Inbakaran 2006), it is well evident that personality is a powerful characteristic of humans that can be used to help predict their preferences in a wide range of domains (Cantador and Fernández-Tobías 2014). For instance, personality has shown to improve recommendations and solve the RS usual problems like the cold-start and data sparsity problems (Dhelim et al. 2021; Feil et al. 2016; Tkalcic and Chen 2015; Tkalcic et al. 2011). And how about the (leisure^1^) tourism domain? Is personality related to tourist preferences? As well observed by Jackson and Inbakaran (2006), “personality is one of the best known, and potentially the most useful, psychological concepts in tourism.” In the case of recommendations to groups, correlating the users’ personalities and their preferences can help match users with similar interests, minimizing the groups’ heterogeneity and conflicts of interest in (occasional) groups of tourists.

Several studies on the relationship between personality and tourist preferences exist; however, the ones available only focus on specific types of traveling, tourist roles or tourism information search (Delic et al. 2016; Eachus 2004; Ismailov 2017; Jani 2014b; Masiero et al. 2019; Poon and Huang 2017; Schneider and Vogt 2012; Sertkan et al. 2020; Tan and Tang 2013), or mainly target the extraversion and openness to experience personality dimensions (Bujisic et al. 2015; Li et al. 2015). So, do all personality dimensions influence the choice of tourist attractions? What is the relationship between the tourists’ personality and their preferred tourist attractions? Our previous work showed the proposed tourism categories could be predicted by all the personality dimensions (Alves et al. 2020), but since it was an ongoing study, more responses were still being obtained for future analysis, being the work here presented the continuation of that work.

The correlation between the tourists’ personality and preferred tourist attractions can be valuable to automatically model their profile in (G)RS for tourism, solving the cold-start problem and eliminating the need for users to fill long and tedious questionnaires and configurations (Dhelim et al. 2021; Gretzel et al. 2006; Tkalcic et al. 2011). But could personality help find and automatically model other characteristics of tourists, such as their motivations, and travel-related preferences and concerns when traveling in leisure? Can it be used to mitigate conflicting preferences in groups of tourists?

Motivation has long been studied in the area of leisure and tourism (Cohen 1979; Gnoth 1997; Norman et al. 2001; Pearce and Caltabiano 1983; Plog 1974; Vigolo 2017) and is the need that drives someone to travel to a certain destination, or choose a certain tourism activity (Gee et al. 1984; Park and Yoon 2009). Motivation is a complex topic (Crompton 1979) and the “starting point for studying tourist behavior” (Pearce and Lee 2005), being a challenge to study due to the diversity of human needs and cultures (Smith 2014). Tourism offers and tourism marketing strategies greatly depend on those motivations (Gnoth 1997; Lo et al. 2004; Nasolomampionona 2014), existing many motives that can influence someone to choose a destination/activity, such as to relax, explore the destination and more. But are these motives a consequence of the tourist’s personality? Is there a relationship between the Big Five personality dimensions and motivations for traveling? Based on Pearce and Lee (2005) proposed travel motives, we tried to discover if the tourists’ personality could also predict their travel motivations.

But what drives someone to travel may be conditioned by certain travel-related preferences and/or concerns (Alves et al. 2019; Çakar 2020; Hung et al. 2016; Morar et al. 2021). For example, Matthew wants to visit Egypt, but because he cannot go with his girlfriend, he does not feel motivated to go, and so he won’t go unless she does. Or suppose someone is in an excursion in Portugal and the group is going to visit Clérigos Tower, but one of the members is afraid of heights and she doesn’t want to visit it. Although tourist attractions preference and motivations for traveling are important for (G)RS to present a more tailored tourism offer, travel-related preferences and concerns play an important role in the decision for visiting or traveling to a destination. Therefore, we considered important to study that four travel aspects and discover how/if they are influenced by the tourists’ personality.

In short, this study aims to find which tourists’ travel aspects can be predicted by their personality, namely if the choice of tourist attractions, the motivations for traveling, travel-related preferences and concerns are influenced by the tourists’ personality (the Big Five personality dimensions), and help tourism GRS researchers automatically model the tourists profile based on their personality without the need for long initial configurations or the users continuous interaction, and therefore help in the cold-start problem, and how those travel aspects, along with personality, can be used to solve conflicting preferences in groups.

Thus, in 2019 (Alves et al. 2020), we started a large-scale study to determine that relationship. To accomplish that, an intensive research on the four travel aspects was conducted so that a questionnaire to collect as much information as possible could be constructed and disseminated. This paper continues that work, exploring the dataset in more detail, having obtained 555 more responses, in a total of *n* = 1035 viable responses. The Exploratory Factor Analysis (EFA) and Confirmatory Factor Analysis (CFA) for extracting and confirming the proposed tourist attractions categories were improved, corroborating the preliminary work (Alves et al. 2020), showing all personality dimensions were predictors of different preferences for tourist attractions. EFA and CFA were then extended to the items associated with the travel motivations, the travel-related preferences and concerns, and related to the Big Five, having found that travel-related preferences and concerns can be predicted by different personality dimensions, but interestingly, only neuroticism and openness to experience were found to be related to the traveling motivations.

As a final result, to help in the cold-start problem, we propose three models that relate the Big Five personality dimensions to a wide range of tourist attractions, traveling motivations, and travel-related preferences and concerns, and a fourth model, to solve conflicting preferences in groups by creating subgroups of tourists with similar interests. The outcomes are then discussed and evaluated.

The remainder of the paper is structured as follows: Sect. 2 describes the background and some related work on GRS for Tourism and Psychology of Tourism; Sect. 3 presents the methodology used; Sect. 4 the results and their respective analysis, along with the proposed models; Sect. 5 the proposed models formulations that relate personality with preferences for tourist attractions, traveling motivations, and travel-related preferences and concerns, as well as the algorithms to create subgroups of similar interests; and finally, Sect. 6 reflects on the contents addressed in the paper and describes what will be done as future work.

## Background and related work

### Group recommender systems for tourism and group decision-making

To overcome the challenges that (especially casual) groups of tourists bring and to provide a satisfactory list of recommendations are the main concerns of GRS for tourism. Some prototypes of GRS for tourism can be found in literature, like the chat-based GRS of Nguyen and Ricci (2018) where the system learns from the tourists comments and classifications in the chat, and from the rating of previously visited Points of Interest (POI), to provide a list of recommendations. The tourists then need to reach a consensus on the recommended POI to visit via the chat. This can be a complex task depending on the group’s heterogeneity and size. Therefore, the decision-making process is intimately associated with GRS (Delić et al. 2020), as the users in a GRS need to negotiate and reach a consensus on the list of recommended POI to visit (Jameson et al. 2003). Hence, Group Decision-Making (GDM) techniques could help in that part of the process, such as to reach a faster and more satisfying consensus, as no one wants to feel unsatisfied when going for an excursion, dissatisfaction that can easily spread to the group due to the emotional contagion phenomenon (Delic and Masthoff 2018). There are already GRS that consider GDM as a factor of success in the decision process (Alves et al. 2019), such as the GRS proposed by McCarthy et al. (2006), Castro et al. (2015) or Marques et al. (2016). For example, Abolghasemi et al. (2022a, b) proposed a GRS that uses GDM techniques like pairwise scoring methods (Abolghasemi et al. 2022a, b; Herrera-Viedma et al. 2007), and the Thomas–Kilmann Conflict MODE Instrument (TKI) (Thomas 2008), which evaluates how someone behaves in a conflict situation, to simulate the users’ conflict style in the decision process, as the decision is strongly dependent on the group members personality and conflict solving capabilities.

The consideration of GDM techniques can be the leverage needed to reach a consensus on the final recommendations list, and will be further studied in future works. Predicting the users’ preferences based on their personality is the beginning of that path.

### Psychology of tourism

The increased interest on psychology of tourism is undeniable, since it is evidenced that psychological aspects are related to the choice of specific destinations (Jafari 1987; Passafaro et al. 2015; Plog 1974). But which ones? Several researchers tried to answer that question, some by proposing tourist typologies or roles based on psychological aspects, others by trying to find relationships between personality and tourist behaviors or preferences.

#### Tourist typologies/roles

Many tourist typologies/roles can be found in literature and represent the role played by tourists while experiencing a destination (Yiannakis and Gibson 1992). Cohen (1972) was one of the first researchers to propose a tourist typology, composed of four types: the organized mass tourist (least adventurous, more organized and prefers familiarity to novelty), the individual mass tourist (has a certain control over his time and itinerary and is not bound to a group), the explorer (trip self-arranged, likes to meet locals and speak their language without totally immersing herself) and the drifter (extremely independent, likes novelty at maximum and familiarization at minimum).

Plog is another renowned researcher who studied the psychology of travel in tourism (Plog 2001, 1974, 1994). He proposed two main psychographic dimensions, in the opposite extremes of a normally distributed continuum, to characterize the tourists’ travel behavior: Allocentrics, who are more nature related, adventuresome, curious, outgoing, self-confident, seek for novelty and new experiences, and Psychocentrics, who are self-inhibited, anxious, non-adventuresome, prefer the familiar in travel destinations, especially if they can drive to them, and places where they can relax.

Jackson et al. (2001) proposed four types of tourists: the explorer, the adventurer, the guided and the groupie, combining the orthogonal scales of Allocentrics-Psychocentrics and Introversion–Extraversion.

Eachus (2004) proposed a Holiday Preferences Scale by modifying both Plog’s and Jackson et al. (2001) typologies: Adventurous preference, Beach preference, Cultural preference, and Indulgent preference. They found that tourists with high scores in sensation seeking tended to prefer Adventurous and Beach holidays but not Indulgent holidays, and older tourists were more likely to prefer Cultural holidays than younger.

Based on Cohen’s individual mass tourist type (Cohen 1972), Wickens (2002) proposed five micro-types of tourists: the Cultural Heritage, the Raver, the Shirley Valentine and the Lord Byron type.

To enhance the relevance of recommendations in RS, Gretzel et al. (2004) proposed 12 travel personalities and studied how they related to 17 travel activities in Northern Indiana, having found strong correlations between them. The most selected travel personalities were All Arounder, Sight Seeker and Culture Creature. Concerning the relationships found, as also later verified by Delic et al. (2016), most travel personalities were related to multiple activities; for instance, Shopping Sharks type was related to tourists more interested in shopping, nightlife, and dining. Culture Creatures preferred festivals, museums, and historic sites. Family Guy was not related to gambling, biking, or hunting/fishing. Trail Trekkers were less related to City Slicker, Shopping Shark, and Gamer travel types. Boaters did not consider themselves as Sight Seekers, and Beach Bums did not identify with the History Buff category. The other types corresponded to their respective activities. Later, the same authors studied if the proposed travel personalities could predict the activities and/or places to be recommended by a destination RS (Gretzel et al. 2006), finding travel personalities are “very good proxies for capturing user personality traits and preferences and can be used to make specific destination recommendations” (Gretzel et al. 2006).

As pointed by Gretzel et al. (2006), “it is not clear how easy it is for individuals to select and identify with an existing” typology or how they can actually predict the tourists’ behavior. Although being a potential way of describing types of tourists and creating marketing segments, typologies do not allow to understand what personality dimensions and/or traits are behind those preferences, and therefore are not easy to implement in a (G)RS without the need of certain initial configurations by the user, problem we propose to mitigate by automatically predicting the tourists’ preferences for tourist attractions based on their personality.

##### Personality as predictor of preferences for tourist attractions/destinations

As pointed by several authors, the existing research on tourism behavior is mostly descriptive instead of predictive (Jackson et al. 2001; Schneider and Vogt 2012) which is a limitation that needs to be overcome, i.e., what personality dimensions or traits are predictive of the tourists’ typologies or behaviors/preferences found in literature?

Crotts (1990) found the more dogmatic (close-minded) the participants were, the less novelty and more familiarity they wanted in their vacations, and the ones that had a greater need for cognition, and tendency to engage in and enjoy thinking sought more for novelty.

According to Bujisic et al. (2015), individuals with higher level of openness to experience tended to be more satisfied with aesthetic and escapist experiences than those with lower level. In contrast, individuals with lower openness to experience were more satisfied with entertainment and educational experiences compared to the ones with higher level. Extroverts tended to be more satisfied with educational and escapist experiences.

Although many studies on psychology of tourism for different tourism sectors can be found, many are about typologies of tourists (Addison 1999; Lipscombe 1995; Millington et al. 2001; Plog 2002), which as mentioned before, are descriptive of the tourists’ behavior and do not predict how that behavior influences the choice of tourist preferences. Others try to predict how psychological aspects influence tourist behavior or preferences, but most of them only rely on Sensation Seeking, extraversion, and/or openness to experience scales (Bujisic et al. 2015; Jackson et al. 2001; Li et al. 2015; Nickerson and Ellis 1991), which do not cover all Big Five’s dimensions. Few studies try to correlate all Big Five dimensions with tourist behaviors or preferences. For example, Neidhardt et al. (2014, 2015) performed a factor analysis on the 17 tourist roles proposed by Gibson and Yiannakis (2002) and the Big Five personality dimensions, obtaining seven factors that captured the tourists behavior, where some of them revealed to be correlated with personality dimensions: (1) *Sun loving and connected*—highly correlated with the sun lover tourist role and the neuroticism, openness and conscientiousness personality dimensions; (2) *Educational*—correlates organized mass tourist and educational tourist with agreeableness; (3) *Independent*—combines independent mass tourists I and II and seeker; (4) *Culture loving*—correlates archeologist and high-class tourist with extraversion; (5) *Open minded and sportive*—combines anthropologist and sport tourist with extraversion; (6) *Risk seeking*—results from the correlation of action seeker, explorer and jetsetter; (7) *Nature and silence loving*—correlates escapist I and II.

Kvasova (2015) studied how personality influenced tourists’ eco-friendly behavior, finding individuals with high agreeableness were strongly related to eco-friendly behavior, followed by conscientiousness and neuroticism, confirming several past studies on the same area of research (Hirsh 2010; Markowitz et al. 2012; Milfont and Sibley 2012). Regarding openness to experience, individuals with high imagination were negatively associated with eco-friendliness, but individuals with high intellect were positively associated.

Jani (2014b) and Delic et al. (2016) studied how the Big Five correlated with a variety of tourist roles. Jani (2014b) explored that relationship using the Big Five and the 12 travel personalities (types) proposed by Gretzel et al. (2004). The author found significant personality differences between the travel types. Those who enjoy games of any type (Athlete type), historical sites, shopping and water activities/attractions (Boater) are high in openness to experience, and those who like to lay around the beach (Beach bum) and spend time with family are low in that dimension. Shopping and Family travel types have a high conscientiousness, and Athlete and Gamer types are low in that factor. Trekker and All things travel types have higher extraversion, and Cultural, Beach bum and Boater types are lower in extraversion. As for high agreeableness, it includes Boater and Family travel types, and low agreeableness the Gamer type. Low neuroticism was associated with Family and All things travel types. Delic et al. (2016) analyzed the relationship between the 17 tourist roles defined by Gibson and Yiannakis (2002) and the Big Five. For example, Sun Lover type was related to high neuroticism individuals, Archeologist to extraverts and Drifter to less conscientious people. No significant correlations were found between the other tourist roles. As expected, they also found tourist roles varied with age, but that the Big Five personality dimensions were stable across ages.

All these studies show that the travel behavior and preferences are related to the tourists’ personality. However, none, to the best of our knowledge, correlates the Big Five personality factors to the choice of raw categories of tourist attractions, but instead, to tourist typologies. With this work, we intend to fill that gap by proposing a model to predict the preference for a wide range of tourist attractions, adapted from the “Thesaurus on Tourism and Leisure Activities” of the World Tourism Organization (Organization 2001), based on the tourists’ five personality dimensions, aiming to help tourism (G)RS to provide recommendations for visiting attractions/destinations just by knowing the tourist’s personality and solve problems related to conflicting preferences in (occasional excursion) groups. We believe that creating subgroups with similar personalities, and therefore, similar tourist preferences, can help solve those conflicts.

This research is motivated by the evidence found in literature, from which it is possible to reason that the tourist typologies do not fully justify the tourists’ preferences for tourist attractions, since many different combinations of intensity for the personality traits exist, and therefore, a single typology may not be enough for a certain tourist and not all the attractions present in a typology may be suitable for that tourist. This claim is supported by the results found by Gretzel et al. (2004) and Neidhardt et al. (2015). Although it is “easy” to recommend attractions based on a single personality dimension, individuals have a combination and different scores on the five personality dimensions. How do we aggregate all that to recommend the right tourist attractions? We cannot recommend an attraction classified for high extraversion and low neuroticism to someone low in both dimensions.

#### Tourism motivation

Many studies on tourism motivation exist, some studying motives for traveling to specific sites (Collins-Kreiner and Kliot 2000), tourism niches (Hassani and Moghavvemi 2019; Heung and Leong 2006; Otoo and Kim 2020), senior tourists (Boksberger and Laesser 2008; Otoo et al. 2021; Patuelli and Nijkamp 2016; Vigolo 2017), or in general (Heitmann 2011), others to propose scales or dimensions of motivations (Crompton 1979; Pearce and Caltabiano 1983), among others. These studies are particularly important to tourism marketing, and therefore to tourism (G)RS, so better and more tailored services and products can be delivered to tourists.

One of the first researchers to care for the tourists’ motivations was Dann (1977), by trying to answer the question “What makes tourists travel?”. Although some viewpoints could be found at the time, claiming the major reason for traveling was to escape from the daily routine, the ordinary, etc., there was no empirical evidence to demonstrate it (Dann 1977; Lundberg 1971). However, a general classification to explain tourist motivation with “push” and “pull” factors was widely accepted (Dann 1977; Heitmann 2011). “Push” factors refer to the tourist’s physiological and psychological aspects (e.g., escape, relax, etc.) influencing his decision to travel, like needs and preferences. “Pull” factors pertain to the characteristics of the travel destination or external motivations that attract (pull) the tourist to visit it.

Later, Iso-Ahola (1982), suggested tourism motivation was mainly driven by escape and seeking, both having personal (psychological) and interpersonal (social) factors, and therefore, he distinguished four dimensions: personal seeking, personal escape, interpersonal seeking and interpersonal escape.

McIntosh and Goeldner (1985) proposed five types of motivations, reflecting the ideas of the Maslow’s pyramid: Physical (the need for relaxation or other activities to reduce stress or refresh the body and mind), Emotional (to seek romance, adventure, spirituality, escapism or nostalgia), Cultural (to learn about the destination’s culture and heritage), Interpersonal (the need to maintain or develop new relationships, by visiting relatives or friends, or meet new people), and Status and prestige (the need to enhance self-status and receive attention/valorization from others).

A very interesting travel motivation theory was developed by Pearce (1993), Pearce and Caltabiano (1983), and Moscardo and Pearce (1986): the Travel Career Ladder (TCL), latter modified to Travel Career Pattern (TCP) since tourists could be at more than one level at a time (Pearce and Lee 2005). Also based on the Maslow’s needs hierarchy (Maslow 1970), the theory describes five different levels of tourist needs, from bottom to top: relaxation needs, safety/security needs, relationship needs, self-esteem and development needs, and finally, self-actualization/fulfillment needs. The theory argues that tourists’ motivation changes according to their age and/or travel experience, resulting in a travel career. To understand pleasure travel motivation more broadly, Pearce and Lee (2005) identified a wide range of travel motive items and determined 14 underlying motivation factors: Novelty, Escape/relax, Relationship (strengthen), Autonomy, Nature seeking, Self-development (host-site involvement), Stimulation, Self-development (personal development), Relationship (security), Self-actualize, Isolation, Nostalgia, Romance and Recognition. They found escape/relax, novelty, relationship and self-development were the most important motives for traveling. The more experienced travelers were more motivated by self-development through host-site involvement and nature seeking. The low experienced were more driven by stimulation, personal development, self-actualization, security, nostalgia, romance and recognition.

Literature on travel motivation is very extensive, and therefore, only some works were presented, but one thing is clear; the main reasons for traveling have been very similar in the last decades and among different age echelons, being Exploration, to have Cultural/Nature experiences, and Relaxation/Escapism the most common motives.

##### Personality as predictor of tourism motivation

By analyzing why someone chooses to travel to a specific site or tourist attraction can help find their traveling and behavioral patterns, which can greatly help improve the tourist’s profile in a (G)RS and thus provide better recommendations. And, if, for instance, personality could be related to the motives behind traveling, it would be easier to propose certain attractions or destinations by just knowing the tourist’s personality. As Heitmann (2011) points out, many factors can influence the tourists’ behavior and choices, such as cultural and religious factors, demographics, and personal factors such as personality, lifestyle, occupation, income, among others. So, how does personality relate to the most common tourists’ motivations?

As mentioned in Sect. 2.2, several tourist roles and typologies have been proposed to describe tourist behaviors (Cohen 1972; Gray 1970; Plog 1974; Smith 2012), but they do not explain the reasons behind those behaviors (Heitmann 2011).

Not many works that study the relationship between (Big Five) personality and traveling motivations were found, and the ones existing, to the best of our knowledge, aim to relate personality and the motivations for specific tourism niches or destinations, such as religious tourism and cruise ship tourists (Abbate and Di Nuovo 2013; Scaffidi Abbate et al. 2017), travel curiosity (Jani 2014a; Kashdan et al. 2009), volunteer tourism (Suhud 2015) or just for the travel desire (Labbe 2016), or relate other personality types to the travel intention (Kaewumpai 2017; Kim et al. 2019; Kwon and Park 2015; Otoo et al. 2021), or only to one motive. Others use different scales of personality (not the Big Five).

A recent study of Scaffidi Abbate et al. (2017) compared the motivations (*Curiosity and discovery*, *Out-of-routine* and *Self and sociality*) and personality of religious travelers versus cruise ship tourists. Regarding religious travelers, the authors found openness to experience positively predicted *Curiosity and discovery* motivation and agreeableness negatively. Agreeableness and conscientiousness negatively predicted *Out-of-routine* motivation. *Self and sociality* was predicted by negative scores in openness to experience. A different pattern was found in cruise tourists, where openness to experience and agreeableness both positively influenced the curiosity motivation, and conscientiousness negatively. *Out-of-routine* motivation was negatively predicted by conscientiousness and neuroticism. Finally, openness to experience, extraversion (energy) and conscientiousness positively predicted *Self and sociality* motivation.

The findings in literature show that certain motivations for traveling in specific contexts can be predicted by personality dimensions. With this work, we intend to verify if that applies to a greater range of travel motivations, including the most common ones, abstracted from specific niches or destinations, and to propose a model to predict tourism motivations based on the tourists’ Big Five personality dimensions.

#### Travel-related preferences and concerns

To choose a travel destination is part of a process that starts with the need/desire for traveling (Mathieson and Wall 1982), and the information that is collected is evaluated according to the traveler’s needs and preferences as well as possible constraints. According to Hung et al. (2016), there are three types of travel constraints: intrapersonal (e.g., to feel guilty for traveling, to be afraid of traveling to a specific destination, limited knowledge of tourism), interpersonal (e.g., lack of travel partners) and structural (e.g., lack of time or money). For instance, many people would like to visit Ukraine, but due to the actual war it is not a choice. Also, someone might prefer to visit a country on summer instead of another season. Or someone may not be able to travel due to money or time constraints. In this study, we focused in the intrapersonal and interpersonal constraints and will consider them as concerns from now on.

Some concerns have shown to intensify with age (Fleischer and Pizam 2002; Lindqvist and Björk 2000; Vigolo 2017; You and O'Leary 1999), like the fear of becoming ill, lack of doctor availability, concerns for safety and personal security, sanitation, service and food quality (Kim et al. 2003; Lindqvist and Björk 2000; Torres and Skillicorn 2004). Health problems are more noticeable in older tourists (> 64 years old), reducing the length of vacations (Fleischer and Pizam 2002), and increasing the concerns about traveling to long-haul or less developed destinations, flight durations, health insurance or even humidity (Hunter‐Jones and Blackburn 2007). As pointed by Vigolo (2017), Huang and Tsai (2003) found senior Taiwanese travelers revealed preoccupation for leaving their house unattended, not having travel companions, dietary restrictions, or not having an enjoyable time and waste money. Chinese women were more concerned about “limited knowledge of tourism, health and safety, culture shock, lack of travel partners, low-quality service facilities, limited availability of information and negative reputation of tour guide” (Gao and Kerstetter 2016; Vigolo 2017). Emotional barriers like fear of the unknown, loss of freedom and loss of spontaneity were pointed as the highest barriers for family caregivers and their care-recipients by Gladwell and Bedini (2004).

Although safety and security have long been key concerns for many tourists (Larsen et al. 2009; Poon and Adams 2000), tourism in general is not seen as risky (Sönmez and Graefe 1998a, b). However, certain unexpected and tragical events can decrease the tourists’ confidence and reduce the desire to travel. The attacks on the World Trade Center and the Pentagon, on September 11, 2001, were a sad example, which led to the mass cancelation of inbound and outbound flights (Floyd et al. 2004). The actual COVID-19 pandemic is another case, where to travel, either overseas or within the same country, is considered risky, and was even forbidden to many countries (Borkowski et al. 2021; Godovykh et al. 2021; Morar et al. 2021; Neuburger and Egger 2021; Tabak et al. 2021; Zenker et al. 2021).

The study of the perceived risks in tourism has long been investigated (Dolnicar 2005), being the concept first introduced by Bauer (1960). According to Dolnicar (2005), the study of perceived risks can be classified into two dimensions: negative perceived risks, which are not sought by the tourist, and positive perceived risks, which are actively sought by the tourist, such as sensation seeking activities. In their investigation of the fears Australian tourists associate to leisure travel, in the context of domestic and overseas travel, Dolnicar (2005) found five categories of risk factors: (1) political risk, such as “terrorism, political instability, war/military conflict”; (2) environmental risk, like “natural disasters, landslides”; (3) health risk, like “lack of access to health care, life threatening diseases, lack of access to clean food and water”; (4) planning risk, such as “unreliable airline, inexperienced operator, not assured flight home”; and (5) property risk, such as “theft, loss of luggage”. All the referred risks were more frequently associated with overseas travel. As for domestic travel, wildlife and the road’s condition were the greatest concerns. The context may change everything. Negative events in association to fears and concerns can prevent the tourist from visiting certain places/attractions, from being involved in particular activities, or even from traveling.

As for travel-related preferences, someone might prefer to travel accompanied or alone, to a cold or hot weather destination, or to take a flight or travel by car, and so on. Travel preferences can also influence which destination to visit or even the decision to travel at all. For instance, Otoo et al. (2020) studied eight travel-related features/preferences: travel duration (by flight), travel partners, accommodation type, travel arrangement type (own or package tour), information technology acceptance, tourism type (e.g., urban, eco, health), attractions type (e.g., historical, natural scenery) and activities type (outdoor, shopping, dining), and related them to the travel motivations they found. Ramires et al. (2018) studied what travel preferences and destination attributes tourists visiting Porto in Portugal preferred, namely travel organizer, travel partners, transport to destination, type of accommodation, type of activities in the destination, transport in the destination and how they were related to their travel motivations.

Many travel preferences and concerns can influence the travel plans, bringing limitations or even prevent tourists from traveling. To know them can improve (G)RS recommendations. But how does personality fit in these all? Is it an influencing factor for those preferences and/or concerns?

##### Personality as predictor of travel-related preferences and concerns

Many studies that relate tourist typologies or personality to travel-related preferences and/or concerns, especially concerns, could be found.

Sandra Lee Basala (1997); Sandra L Basala and Klenosky (2001) found individuals with different travel styles (Familiarity Seekers, Average Travelers, and Novelty Seekers) had different travel-related preferences, namely regarding the type of accommodations, type of travel companions and language of the host destination.

Beside relating demographics, Jackson and Inbakaran (2006) studied tourists visiting Australia and how their proposed personality types (Explorer: introvert + allocentric, Adventurer: extravert + allocentric, Guided: psychocentric + introvert, and Groupie: psychocentric + extravert) related to the preference for pre-planning a vacation, using internet to book travels, traveling alone, travel companions, intention to revisit a destination, length of stay and destination’s cultural similarity.

Considering three personality dimensions (extraversion, conscientiousness, and emotional sensitivity (neuroticism)), Maritz et al. (2013) studied how tourists’ personality influenced the perceived travel risks (personal risk, property risk and liability risk), the traveling intention, and the perceived risk on travel intention. As for perceived travel risk, conscientiousness and emotional sensitivity positively influenced personal risk, emotional sensitivity positively predicted property risk, and finally, all three personality dimensions showed positive effects on liability risk. Extraversion did not appear to be affected by travel risks. Regarding travel intentions, all three personality dimensions positively influenced the travel intention. Perceived risk significantly affected travel intention in terms of personal and liability risks. Evidence suggested the perceived risk would reduce the influence of extraversion, conscientiousness and emotional sensitivity on travel intention.

Using Plog’s psychographic model (Plog 2002), Morakabati and Kapuściński (2016) focused on the relationship between risk perception and the destinations’ benefits, and if terrorism affected the willingness to travel according to the tourists’ personality. Yazdanpanah and Hosseinlou (2016) studied the influence of the Big Five personality dimensions on the choice of transportation mode according to the weather conditions.

Carvalho et al. (2020) studied if extraversion and conscientiousness were related to social distancing and handwashing COVID-19 containment measures in Brazil. They verified the less extraverted the more concerned with social distancing the participants were. Participants who considered neither of the two containment measures had lower conscientiousness scores. Participants that adhered to both or one of the containment measures had higher conscientiousness.

Another study on COVID-19 pandemic (Aschwanden et al. 2021), focused on concerns related to the pandemic (e.g., become sick with coronavirus), precautions to avoid catching the disease (e.g., wear face mask), preparatory behaviors (e.g., stockpiling food) and duration estimates concerning the disease (e.g., time to return to normality), showed high neuroticism was related to more concerns but to fewer precautions, and unrelated to preparatory behaviors; high conscientiousness to more precautions; higher scores on extraversion were predictors of more optimistic duration estimates; and higher neuroticism of more pessimistic ones. Age showed to moderate the personality effect, revealing to be a great predictor of psychological and behavioral responses to the disease, especially in older adults (aged 65 +): greater concerns were a result of higher openness scores, high openness and agreeableness values were predictors of more preparations and higher duration estimates, higher conscientiousness was positively associated with more preparatory behaviors, but had non-significance for middle-aged (40–64 years) and younger adults (18–39 years old), but was related to shorter-duration estimates in middle-aged and younger adults and higher duration estimates on older adults. A similar study was also performed by Al-Omiri et al. (2021).

Faullant et al. (2011) studied how extraversion and neuroticism influenced the joy and fear basic emotions in a mountaineering experience and in the satisfaction formation. They confirmed their proposed hypothesis that extraversion positively predicted joy, and neuroticism positively predicted fear, i.e., the more extraverted mountaineers experienced higher levels of joy while the more neurotic ones were more susceptible to experience fear.

It is undeniable that some leisure activities are more pleasant, or only possible, under certain weather conditions. For instance, to relax on the beach is more enjoyable in a sunny and warm weather than on cold or rainy conditions (Sabir 2011; Shi 2012); skiing is only possible on snowy conditions (not considering artificial snow). Liu et al. (2021) verified both tourists with low- and high-place attachment greatly diminished their intentions to visit a National Forest in Taiwan when negative climate changes occurred. These are in line with the observation made by Shi (2012) that many tourists are motivated for traveling on particular weather conditions, selecting times of the year where the climate conditions are more favorable to them. The thermal comfort “has a decisive influence on national and international tourist flows, and largely controls the duration of the tourist season, especially in mid- and high-latitude regions” (Mieczkowski 1985). It is evident that the seasons and/or climatic conditions have a psychological effect on the tourists’ motivation to travel (Becken 2010; Scott and Lemieux 2009), but is personality an influencing factor?

Besides the ones presented, many other variables can influence travel preferences and concerns, like the cases of certain phobias such as the fear of heights, dark, confined spaces, reptiles, among others. It would be very bad if a (G)RS recommended a tourist to visit the Eiffel Tower if she was afraid of heights, or to play an escape game with a dark and confined spaces theme. And where is personality in the middle of all those phobias? Mellstrom et al. (1976) argued individuals scoring high on thrill and adventure seeking were less prone to feel fear in anxiety-inducing situations; in fact, they might feel attracted to those situations. In their study, female students who were more anxious and neurotic revealed more fear of snakes, heights and darkness.

As pointed by Morar et al. (2021), “it is well recognized that personality plays a special role in both perceptions of risks and preferences related to travel.” However, most of the studies found were related to travel risks and perceptions of risk, and travel-related preferences. Studies correlating personality to other types of concerns, such as phobias, or climate preferences, were very hard to find. With this study, we hope to fill those gaps and provide a more thorough and complete study on how the Big Five personality dimensions impact travel-related preferences and concerns.

## Methodology

This work continues the work presented in Alves et al. (2019, 2020). The same online questionnaire,^2^ created using Google Forms, was used to gather extra responses, since we wanted to increase the sample’s heterogeneity and improve the “Personality vs Tourist Attractions Preference” model fit, as some of the limitations found were the sample’s size and that most of the respondents had higher education, specially from Exact and Social Sciences. Using again the snowball sampling method, the questionnaire was sent, through a link using an especially elaborated email, to adolescents and adults (15 + years old) in Portugal with more diverse backgrounds and education/areas of formation, like the parents/sponsors of education of students and the educational community of two elementary and high-schools; scholars, professors and general employees of the Faculty of Fine Arts of the University of Porto and Lusófona University; to the Geek Girls Portugal group; and Herbalife® Group members. The link was also posted in two Facebook® groups (Association of Portuguese Research Fellows, and Travels Around the World Portuguese group). This resulted in 545 additional responses, in a total of *n* = 1063 responses (summed up to the 518 responses obtained in the previous study), collected from January to September 2020.

The aggregated sample was then cleaned for inconsistent responses and several univariate outliers were removed (Pyle 1999; Witten et al. 2016), using both Microsoft® Excel® 365 and IBM® SPSS® Statistics 26. Namely, 16 responses were removed due to duplicated data, dubious, incoherent or playful responses. Since responses in an extreme do not really mean an outlier behavior, the sample was searched for unengaged respondents, namely respondents who entered repeating patterns in questionnaire sections with Likert scales, like entering only “7,7,7,7,7…” or “1,2,3,4,5,1,2...”, etc. Respondents were also examined if they responded normal questions in the same direction as reverse-coded questions (BFI section), for example, “I am talkative” and “I am reserved.” With these methods, 12 more outliers were removed, resulting in a final sample of *n* = 1035. Boxplots were also drawn in SPSS for each variable, but as the sample was not very large, the outliers found by this method were not removed. Inconsistent open responses were uniformized (socio-demographic questions, section I), for example, some respondents answered in “country of birth” variants of “Portugal”, like “Portuga” or “Portuguesa”, and so, all inconsistent situations were converted to Portugal. There were no missing values in the questionnaire, except for the sensitive questions, which were not mandatory.

Using SPSS, several runs of Exploratory Factor Analysis (EFA) (Fabrigar et al. 1999), an unsupervised machine learning technique, were performed on the BFI (questionnaire section II, 44 items) and on the traveling motivations items (section IV, 28 items), to check if the items aggregated as expected in the original scales, to ensure the unidimensionality and discriminant validity of the scales (Clark and Watson 2016); and on the items of the travel-related preferences and concerns (section III, 34 items), and of the tourist attractions preference (section V, 68 items), to discover latent factors underlying the dataset, and therefore unveil which items had the strongest correlation to a given factor (DiStefano et al. 2009). All four EFA were performed using the principal components extraction method with Varimax rotation and Keiser normalization (Eigenvalue > 1), suppressing coefficients with a saturation below 0.40 in the factors. Items scoring in more than one factor, with a difference less than 0.10, were removed, as well as items with a communality below 0.50 that weren’t contributing to the models adequacy (Bryman and Cramer 1992; Marôco 2010). Factors with low reliability (Cronbach’s Alpha α < 0.60) were also removed (Hair et al. 2009). Except for the BFI scale, the resulting factors were then renamed to more meaningful names, to better represent the concepts they were measuring (see Tables 7, 9 and 11).

After performing the EFA, the Structural Equation Modeling (SEM) and Confirmatory Factor Analysis (CFA) methodologies (Byrne 2016; Marôco 2010) were conducted on each extracted scale, using IBM® SPSS® AMOS version 26. For each scale, a first-order model was drawn to confirm if the observed variables (questionnaire items) saturated the latent variables, i.e., the extracted factors, and what correlations existed between them. For the estimations, the maximum likelihood method was applied. Several adjustments were needed to reach an acceptable/good goodness-of-fit of the models, such as eliminating items with a regression weight lower than 0.50 (Marôco 2010), and covarying errors within the same factors as suggested by the largest modification indexes (maintaining only the ones with statistically significant covariances with values ≥ 0.10 (Marôco 2010)), resulting in the proposed final scales, shown in Table 6 , Figs. 3, 5 and 7.

The resulting BFI scale was then used to predict the preference for the (I) proposed tourist attractions, (II) travel-related preferences and concerns and (III) travel motivations, again using Structural Equation Modeling and CFA, applying the maximum likelihood method for estimation. To improve the models’ fit, the same adjustment process used in the previous CFA was applied. This resulted in the final proposed models: “Personality vs Tourist Attractions Preference” (Fig. 4), “Personality vs Travel Motivations” (Fig. 6) and “Personality vs Travel-related Preferences and Concerns” (Fig. 8).

All the obtained results are detailed in Sect. 4.

## Results and analysis

During the questionnaire dissemination, it was very hard to find the needed respondents, and we can say it was not related to the questionnaire’s size, as we performed other type of questionnaires in other works, much smaller, and the same difficulty was found. Persons are not so available to help and probably they find boring to fill questionnaires, independently of their size.

We noticed respondents had some difficulties in filling the formation area question, as it was an open question due to the heterogeneity inherent to the possible responses. Many respondents did not know what to answer and responded areas that did not correspond to the intended. Extra work was needed to clean the data and make the right correspondence.

### Sample characterization

#### Demographics

**Fig. 1:**
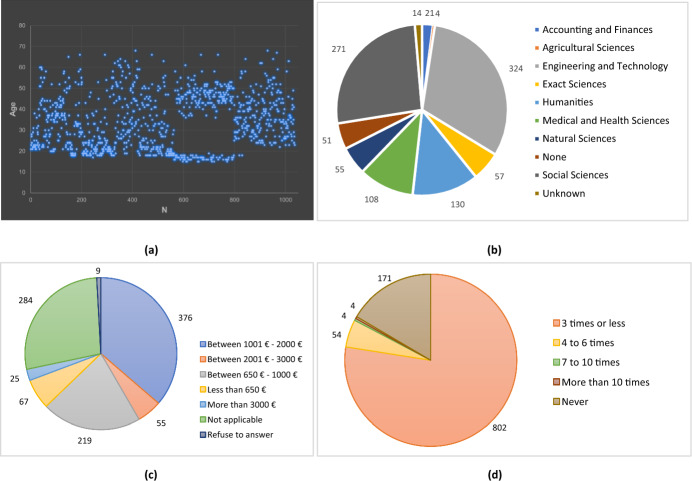
Respondents’ **a** age range, **b** formation areas, **c** liquid income, **d** leisure travels abroad per year; *n* = 1035

As can be seen in Table 2 and Fig. 1, the sample is composed mostly by Portuguese citizens (94%) of a varied age range, being the majority females (74%), adults and young adults (≤ 55 years old, 94%), with 70% between 23 and 55, and a mean of 35 years old. 60% of the respondents are in some sort of a relationship and 42% have children. Most of them live with their partners and/or children (53%), and 36% with their parents and/or other relatives (as 24% of the sample has less than 23 years old and many are still studying). As for the formation area, 31% are from “Engineering & Technology,” 26% from “Social Sciences,” followed by 13% from “Humanities,” 11% from “Exact” and “Natural Sciences,” and 10% from “Medical & Health Sciences,” but only 69% have higher education. Regarding the professional situation, most of the sample represents employed workers (61%, where 8% are self-employed as employer or as isolated), 28% students, 7% working students, and the remaining 4% are unemployed, domestic or retired. 25% of the respondents had already lived in other countries and 97% visited other countries, where 66% traveled to 4 or more countries in their life and 56% to other continents besides Europe, within the last 2 years (79%, 6 months (41%)), meaning they have a richer experience of different cultures. Most of the sample traveled abroad 3 times or less per year (78%), meaning the contact with other cultures, although diverse and recent, is not routinely experienced. When in leisure, most of the respondents (96%) travel accompanied, of which 79% are family.

**Table 2:** Sample descriptive statistics (*n* = 1035)

		*n*	%			*n*	%
Age	< 23	253	24.4	Marital status	Single	365	35.3
	23–35	303	29.3		In a relationship	185	17.9
	36–45	238	23.0		Non-marital partnership	71	6.9
	46–55	188	18.2		Married	360	34.8
	> 55	53	5.1		Divorced/Separated	47	4.5
	Min = 15	Max = 68			Widow(er)	7	0.7
Sex	Female	769	74.3	Has children	No	604	58.4
	Male	266	25.7		Yes	431	41.6
Citizenship	Portuguese	970	93.7	Lived in other Countries	Yes	262	25.3
	Other	65	6.3		No	773	74.7
Higher education				Health problems	None	654	63.2
					Allergies/Intolerances	182	17.6
	No	325	31.4		Chronic disease	99	9.6
	Yes	710	68.6		Disability	16	1.5
					Combination	64	6.2
					Refused to answer	20	1.9
Formation area	Engineering & Technology	324	31.3	Lives with	Alone	87	8.4
	Exact Sciences	57	5.5		Partner and/or Children	546	52.8
	Humanities	130	12.6		Parents/Other relatives	374	36.1
	Medical & Health Sciences	108	10.4		Friends/Colleagues	28	2.7
	Natural Sciences	55	5.3	Professional situation	Employed	627	60.6
	Social Sciences	271	26.2		Student	293	28.3
	None/Unknown	65	6.3		Working student	70	6.8
	Other	25	2.4		Other	45	4.3
Liquid income (after taxes)	Less than 650€	67	6.5	Professes a religion	Yes	493	47.7
	Between 650 and 1000€	219	21.2		No, but believes in a God/Superior Being	230	22.2
	Between 1001 and 2000€	376	36.3		No, is agnostic	128	12.4
	Between 2001 and 3000€	55	5.3		No, is atheist	173	16.7
	More than 3000€	25	2.4		Refused to answer	10	1,0
	Not applicable	284	27.4	Different countries visited	3 or less	320	30.9
	Refused to answer	9	0.9		4 to 10	443	42.8
Continents visited	Europe	437	42.2		11 or more	237	22.9
	Europe, Africa	103	10.0		Never visited other countries	29	2.8
	Europe, Africa, America	128	12.4		Refused to answer	6	0.6
	Europe, America	142	13.7	Last time visited another country	Last 6 months	425	41.1
	Europe, Asia, Africa, America	87	8.4		Last 2 years	389	37.6
	Other combinations	109	11,0		More than 2 years ago	188	18.2
	Never visited other continents	29	2.8		Never	33	3,1
Travel companions	Friends/Colleagues	158	15.3	Travels abroad per year	Never	171	16.5
	Partner	231	22.3		3 times or less	802	77.5
	Partner and children	277	26.8		4 to 6 times	54	5.2
	Relatives	312	30.1		7 to 10 times	4	0.4
	Nobody	41	4.0		More than 10 times	4	0.4
	Other	16	1.5				

Some statistics are not shown as they are related to another ongoing study

Compared to the previous study (Alves et al. 2020), there are more adults between 23 and 55 years old (56% before, now 70%), and the number of respondents with children increased from 31 to 42%, meaning there are more participants supposedly with different responsibility/concerns. The formation areas are also more varied. The other sample characteristics remain similar.

#### Personality

**Fig. 2:**
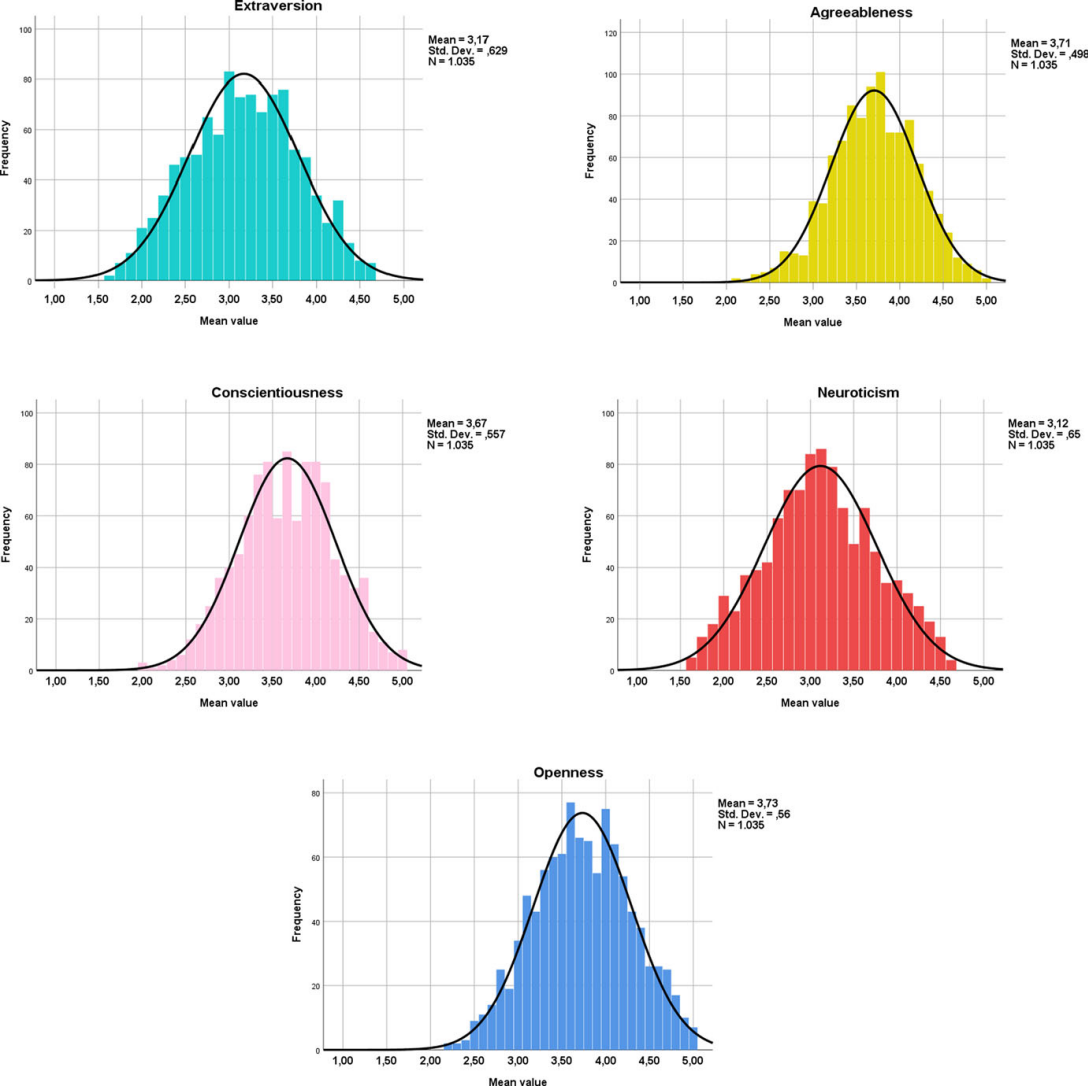
Distribution of the five personality dimensions responses (participants’ mean value)

To assess the respondents’ personality, the Big Five Inventory (44 items) was used, which is one of the most widely used personality inventories. The BFI assesses an individual on the Goldberg’s Big Five dimension of personality (Goldberg 1990), using a 5-point Likert scale. To facilitate interpretation, instead of calculating the scores for each participant, the mean value for each personality dimension is presented. Figure 2 shows the responses distribution for each dimension.

Clearly, there are 3 dimensions with responses above the mid-point, revealing a slight negative skewness, i.e., participants situated themselves more between “3-Neither agree nor disagree” and “5-Agree strongly”: agreeableness, conscientiousness and openness, confirming the results found in our previous study (Alves et al. 2020), reflecting the same social desirability bias, which usually happens more in self-reporting questionnaires (Pedregon et al. 2012), like the desire of being kind and moral in the case of agreeableness; truthful, self-effective and effortful in the case of conscientiousness; and more intellectual in the case of openness to experience. The other two dimensions, extraversion and neuroticism, have the mean value near the scale mid-point.

All five distributions follow the shape of a normal curve, and according to the values of skewness and kurtosis obtained, and respective standard errors, although having a slight skew and kurtosis, they are in acceptable ranges and the data are considered not significantly different from a normal distribution^3^ (Field 2013; Gravetter et al. 2020; Sposito et al. 1983).

#### Tourist attractions preference

A total of 68 items representing a wide range of different tourist attractions, following the most significant terms from the United Nations World Tourism Organization (2001), were presented to the respondents in the questionnaire. Table 3 summarizes the aggregated results.

**Table 3:** Participants' preferences for tourist attractions, in percentage of agreement

		Totally disagree						Totally agree
		1	2	3	4	5	6	7
A1	Go to a Gastronomy Festival (food and/or drinks)	5.7	6.6	7.0	14.1	*24.5*	*22.2*	*19.9*
A2	Watch a natural phenomenon (e.g., volcanic eruption or northern lights)	2.6	2.0	3.5	7.6	*12.7*	*25.9*	***45.7***
A3	Watch a religious celebration	*21.1*	*13.6*	*13.6*	*18.3*	15.9	9.9	7.6
A4	Visit the historic cities/villages of the destination	0.5	1.3	1.7	5.6	*13.1*	*30.6*	***47.1***
A5	Visit an oceanarium	7.6	5.8	8.2	*17.9*	*23.4*	*20.6*	*16.5*
A6	Visit caves/caverns/volcanoes	4.3	3.1	3.4	10.8	*19.1*	*27.5*	*31.8*
A7	Visit archeological sites / ruins	1.4	2.4	3.1	12.0	*20.3*	*26.3*	*34.6*
A8	Attend cultural activities / artistic performances	1.4	1.7	4.3	11.1	*23.3*	*30.6*	*27.5*
A9	Go to the disco/nightclub	*28.3*	*16.0*	*12.9*	*16.0*	13.6	8.3	4.7
A10	Appreciate natural landscapes	0.2	0.3	0.8	3.0	*8.7*	*27.7*	***59.3***
A11	Do hiking / mountaineering	2.7	3.1	5.8	10.0	*24.9*	*24.4*	*29.0*
A12	Practice aquatic sports (e.g., sailing, canoeing, diving, jet skiing)	12.6	10.1	10.6	*17.8*	*16.0*	*15.5*	*17.4*
A13	Go to a theme park (e.g., Disneyland Paris)	6.4	6.8	6.4	10.7	*19.3*	*22.7*	*27.7*
A14	Undergo health and wellness treatments (e.g., hydrotherapy centers, mineral water resorts)	10.6	8.1	9.6	*18.1*	*21.1*	*15.0*	*17.6*
A15	Go to a Zoo	15.6	11.1	7.9	*18.1*	*18.7*	*14.9*	*13.7*
A16	Attend a typical celebration of the destination (e.g., popular celebrations, carnivals, fireworks)	2.4	1.5	3.3	8.9	*19.8*	*31.5*	*32.6*
A17	Go to a film festival	10.3	9.4	12.2	*21.0*	*21.0*	*14.8*	*11.4*
A18	Taste typical local dishes	0.7	1.0	1.6	5.6	*14.0*	*25.7*	***51.4***
A19	Visit a botanical garden	4.5	4.3	5.2	16.4	*24.4*	*23.9*	*21.3*
A20	Visit monuments (e.g., churches, cathedrals, castles, fortresses, monasteries, palaces, etc.)	1.0	1.5	3.4	5.7	*15.1*	*26.9*	***46.5***
A21	Visit a beach for its natural beauty	1.4	0.3	0.8	4.2	*11.0*	*27.0*	***55.5***
A22	Go to the beach (sunbathing/ swimming)	5.4	3.6	4.6	7.9	*17.7*	*21.3*	*39.5*
A23	To enjoy / buy local handicrafts	2.4	2.5	4.2	14.7	*27.0*	*23.8*	*25.5*
A24	Ride a bike	11.3	7.5	8.9	*19.8*	*22.4*	*16.5*	*13.5*
A25	Go to a funfair (e.g., amusements such as Ferris wheel, bumper cars, etc.)	14.0	10.0	9.9	*18.6*	*19.0*	*14.8*	*13.7*
A26	Attend gyms / fitness centers	*44.2*	*17.6*	*13.6*	11.1	7.1	3.9	2.6
A27	Go to a water park	16.9	10.0	11.2	15.9	16.2	13.5	16.2
A28	Go to a SPA / beauty center	*22.1*	*11.6*	*11.6*	*15.7*	14.7	12.0	12.4
A29	Do motorsports (e.g., karting, motocross)	*30.5*	*14.0*	*11.4*	14.8	12.2	8.9	8.2
A30	Have a picnic	6.7	5.6	6.3	17.3	*24.9*	*21.3*	*18.0*
A31	To go shopping / see storefronts (window shopping)	*18.4*	*13.6*	*11.5*	*18.6*	18.7	10.5	8.7
A32	Visit museums of historical themes	3.8	7.3	10.5	9.5	*19.1*	*22.5*	*27.2*
A33	Visit museums of scientific themes (e.g., planetarium, paleontology)	3.8	6.3	10.8	11.2	*19.3*	*24.0*	*24.6*
A34	Visit viewpoints of natural landscape	0.5	2.4	7.0	6.9	*11.5*	*24.7*	***47.1***
A35	Visit viewpoints of urban landscape	3.7	7.5	10.3	14.7	*20.4*	*21.3*	*22.1*
A36	Visit nature or wildlife reserves	1.8	1.3	2.8	7.4	*15.5*	*28.8*	*42.4*
A37	Observe sub-aquatic environments / marine life (e.g., snorkeling, submarine)	10.3	6.2	8.4	14.1	*15.3*	*20.2*	*25.5*
A38	Visit large man-made constructions (e.g., bridges, tunnels, mines)	6.3	6.1	11.3	16.4	*22.1*	*19.5*	*18.3*
A39	Go to a thematic parade (e.g., military, electronic music)	*19.0*	*13.8*	*16.1*	*20.3*	16.3	8.4	6.0
A40	Participate in a gastronomy tour (typical and/or gourmet dishes, wine tasting)	5.9	6.7	8.5	14.0	*24.4*	*20.9*	*19.6*
A41	Walk in the forest / woods	3.3	3.1	3.2	11.2	*24.3*	*28.1*	*26.8*
A42	Take a walk along the river / sea coast	1.1	1.2	2.2	6.3	*20.4*	*32.1*	*36.8*
A43	Go to a music festival/concert	8.5	6.6	9.8	15.3	*21.2*	*21.5*	*17.2*
A44	Go to a dance/ballet festival	15.0	11.4	12.9	18.4	17.6	14.0	10.8
A45	Go to balls (dancing)	*24.6*	*14.3*	*15.7*	*16.7*	12.8	8.2	7.7
A46	Practice climbing or bungee jumping	*30.2*	*13.3*	*13.2*	13.8	13.7	6.5	9.2
A47	Visit mountain areas / gorges	7.4	7.1	10.0	14.1	*21.7*	*20.0*	*19.5*
A48	Go to a live music bar/place	7.7	5.6	9.2	17.4	*23.6*	*21.2*	*15.4*
A49	Take boat trips to know the destination's coast	4.1	5.0	7.4	11.5	*20.4*	*25.0*	*26.6*
A50	Take boat trips for the historical value of the route	5.2	6.5	8.2	13.3	*20.7*	*24.0*	*22.1*
A51	Take boat trips for the pleasure of boating	12.9	10.0	10.8	*14.7*	*15.7*	*18.2*	*17.8*
A52	Take a walk in a city park	1.0	2.0	2.5	11.4	*25.1*	*33.1*	*24.8*
A53	Play ball sports (e.g., football, handball, volleyball, tennis)	*33.6*	*16.4*	*11.6*	13.5	11.3	6.4	7.1
A54	Do a safari	10.0	6.4	7.0	11.5	*20.4*	*19.2*	*25.6*
A55	Play at the casino	***52.2***	*15.1*	*10.8*	9.7	6.4	3.6	2.3
A56	Assist to a sporting competition (e.g., watch a football game from a club of that country)	*34.8*	*12.0*	*10.3*	13.5	13.2	8.7	7.4
A57	Ride a horse	*25.3*	*11.9*	*10.7*	*15.3*	15.7	10.8	10.2
A58	Hunt / fish	***61.4***	*13.2*	*7.5*	7.8	4.3	2.9	2.8
A59	Participate in an escape game	*40.4*	*13.6*	*9.9*	13.7	9.6	6.0	6.9
A60	Watch a bullfight	***82.0***	*6.3*	*3.6*	4.0	2.1	0.8	1.3
A61	Go to the circus	***53.8***	*12.6*	*9.0*	10.4	7.1	3.7	3.5
A62	Go on a cruise	14.4	7.3	6.7	14.6	*17.2*	*17.6*	*22.2*
A63	Do air sports (e.g., parachute jump, skydiving, gliding)	*33.9*	*11.2*	*9.2*	11.8	11.6	10.0	12.4
A64	Go to the swimming pool to swim/dive	11.6	5.4	7.3	13.2	*18.6*	*19.4*	*24.4*
A65	Go to the swimming pool to relax	5.3	4.9	5.6	10.5	*18.8*	*23.1*	*31.7*
A66	Have vacation on an island	3.2	2.3	2.9	8.1	*18.5*	*27.2*	*37.8*
A67	Assist an opera/theater	13.3	7.5	8.4	15.3	*20.1*	*19.2*	*16.1*
A68	Ski	*28.9*	*9.5*	*10.2*	*16.0*	13.6	8.8	12.9

The highest values that contribute to most of the responses are italics, and the values that are similarly distributed are underlined. Values > 45% are in bold

Everyone seems to like almost all sort of tourist attractions when traveling on vacations, with a clear significant majority on attractions like watching a natural phenomenon, visit historic cities/villages, appreciate natural landscapes (including beautiful beaches), taste typical local dishes and visit monuments. The opposite can also be said for attending gyms, playing at the casino, hunting/fishing, (decidedly) watching bullfights and going to the circus, which are definitely not a choice when on vacation. What relationship exist between those preferences and the participants’ personality? Section 4.2.2 shows the results.

#### Traveling motivations

The participants’ traveling motivations were measured using Pearce and Lee (2005) proposed items, using the two items with highest loading for each motive. The items were then mixed up in the questionnaire’s respective section so items from the same motive ended separated. The aggregated results can be found in Table 4.

**Table 4:** Participants' traveling motivations, in percentage of agreement (questions adapted from Pearce and Lee (2005))

		Totally disagree						Totally agree
		1	2	3	4	5	6	7
M1	Being close to nature	1.1	1.1	3.1	9.3	*19.5*	*29.5*	*36.5*
M2	Meeting the locals	1.7	2.4	4.8	12.9	*23.7*	*28.8*	*25.6*
M3	Having daring/adventuresome experience	3.4	3.5	7.6	16.9	*24.3*	*24.7*	*19.6*
M4	Develop my personal interests	0.1	0.3	0.8	5.9	*17.9*	*36.3*	*38.7*
M5	Being with respectful people	0.4	0.3	0.9	8.4	*12.4*	*29.7*	***48.0***
M6	Understanding more about myself	1.4	1.8	2.6	16.3	*18.1*	*26.2*	*33.6*
M7	Being away from the crowds of people	3.3	5.9	9.2	*26.8*	*20.7*	*16.5*	*17.7*
M8	Thinking about good times I’ve had in the past	4.5	6.6	7.4	*23.3*	*18.2*	*19.1*	*20.9*
M9	Having romantic relationships	*25.4*	*13.2*	*7.0*	*20.6*	11.8	11.5	10.5
M10	Showing others I can do it	*22.3*	*13.4*	*11.2*	*23.8*	11.5	9.6	8.2
M11	Feeling the special atmosphere of the vacation destination	0.5	0.6	1.0	6.6	*17.4*	*32.6*	*41.4*
M12	Getting away from everyday psychological stress/pressure	0.5	0.7	1.5	4.3	*9.8*	*25.5*	***57.8***
M13	Doing something with my family/friend(s)	0.3	0.1	0.3	4.8	*11.0*	*27.7*	***55.7***
M14	Being obligated to no one	3.5	3.4	4.0	13.3	*13.9*	*20.6*	*41.4*
M15	Getting a better appreciation of nature	0.7	1.6	1.4	7.6	*16.9*	*28.1*	*43.6*
M16	Observing other people in the area	3.2	3.8	4.7	17.1	*21.2*	*23.5*	*26.6*
M17	Experiencing thrills	3.9	3.9	7.7	*18.6*	*25.6*	*18.3*	*22.0*
M18	Developing my skills and abilities	1.7	1.3	4.0	*14.5*	*21.6*	*26.4*	*30.5*
M19	Being near considerate people	1.3	1.7	2.4	*13.4*	*20.8*	*28.1*	*32.3*
M20	Working on my personal/spiritual values	2.8	2.7	5.0	*20.6*	*19.7*	*24.0*	*25.2*
M21	Enjoying isolation	7.4	10.0	10.5	*23.6*	*20.8*	*14.4*	*13.3*
M22	Reflecting on past memories	11.6	12.4	13.0	26.0	14.6	10.8	11.6
M23	Meeting amorous partners	***53.8***	*15.5*	*5.7*	*13.7*	4.8	3.8	2.7
M24	Being recognized by other people	*23.9*	*11.7*	*11.6*	*21.7*	14.4	8.7	8.0
M25	Experiencing something different	0.9	1.2	1.3	8.6	*20.9*	*28.8*	*38.5*
M26	Getting away from the usual demands of life	0.9	1.1	1.6	6.5	*11.2*	*26.1*	***52.7***
M27	Strengthening relationships with my family/friend(s)	1.4	0.4	1.2	6.7	*14.1*	*28.5*	***47.7***
M28	Being independent	3.8	2.8	3.8	*17.7*	*17.4*	*22.8*	*31.8*

The highest values that contribute to most of the responses are italics, and the values that are similarly distributed are underlined. Values > 45% are in bold

Clearly, except for questions M8 and M22, participants are on the same side regarding motivations for traveling in leisure, confirming most motives proposed by Pearce and Lee (2005). To be close to nature, meet the locals, have adventuresome experiences, develop personal interests and skills, understand more about self & work on personal values, be with respectful persons, get isolated, feel the destination’s atmosphere, experience something different, get away from everyday stress/demands, interact with family/friends & strengthen those relationships, have no obligations & be independent, are all possible motives for traveling in leisure. The great majority also agreed that to meet new amorous partners and get recognized by others were not motives to go on vacation. It is obvious that not all motives are suitable for the same type of vacations and need to be contextualized. And is there a similar personality between similar traveling motives? In Sect. 4.2.3, we analyze how personality relates to those motives.

As previously mentioned, the participants responded quite differently to two questions measuring Pearce and Lee (2005) Nostalgia dimension, M8 and M22. Most of them agreed they want to think about good times spent in the past but are equally divided in reflecting on past memories (37% for agree and disagree). Although scoring in the same Nostalgia dimension, according to the results obtained, M8 is related to thinking about good memories, and M22 relates to past memories, either good or bad, possibly suggesting reflections akin to learning from experience (Table 4).

#### Travel-related preferences and concerns

One section of the questionnaire was related to travel-related preferences and concerns (Alves et al. 2020), where we asked the participants questions related to their preferences and concerns when traveling. The questions and aggregated results are shown in Table 5.

**Table 5:** Participants' travel-related preferences and concerns, in percentage of agreement

		Totally disagree						Totally agree
		1	2	3	4	5	6	7
P1	When traveling on leisure, I prefer outdoor activities	1.1	2.1	4.0	11.0	*22.4*	*30.5*	*28.9*
P2	Under no circumstances I like to take risks related to my physical integrity	3.8	9.3	13.6	*14.4*	*15.8*	*18.4*	*24.7*
P3	A dinner with friends ideally should have a maximum of 6 people	16.3	13.8	12.4	17.5	15.6	13.1	11.3
P4	When going on vacations I take into account the destination's cultural offer	1.5	3.0	5.4	12.5	*16.2*	*29.8*	*31.6*
P5	I am afraid of getting ill or having accidents while away on vacations	11.8	17.6	11.4	13.6	17.2	14.8	13.6
P6	I would never travel to a place where there was no mobile phone network	*21.4*	*18.5*	*14.7*	*14.9*	10.4	10.5	9.6
P7	When planning vacations, I try to include as many places/attractions as possible	2.0	4.9	7.1	13.8	*18.4*	*24.3*	*29.5*
P8	To be perfect, a vacation needs that every day is planned in advance	*14.5*	*16.3*	*17.5*	*15.7*	16.3	12.1	7.5
P9	Within my possibilities, when on a vacation I don't look at expenses	10.8	14.8	18.6	16.4	17.1	13.5	8.8
P10	Before traveling I like to know/study the history of the destination	3.1	5.2	9.6	14.1	*22.9*	*24.2*	*21.0*
P11	Regardless of the destination, it is always better to travel in group	10.9	14.3	14.8	*19.5*	15.6	12.7	12.3
P12	In a distant country, one of my worst fears would be to get lost	12.8	14.6	13.7	13.7	15.0	15.6	14.7
P13	I like to go where few people have been to before	7.7	9.6	11.6	*23.6*	*16.7*	*17.1*	*13.7*
P14	For me, to feel comfort is always the most important (quality of facilities / products)	3.3	7.0	11.7	*17.3*	*29.4*	*19.4*	12.0
P15	For me, to fulfill expectations is more important than a good surprise	8.7	14.1	17.6	25.7	15.2	12.7	6.1
P16	When on vacations abroad, I like to feel that I am contributing to the local economy	8.2	8.2	10.1	30.3	19.2	15.1	8.8
P17	For me, while on vacations there should be no time schedules	3.2	7.1	11.9	14.9	*18.4*	*20.3*	*24.3*
P18	For me, a good vacation has to include a cultural / learning component	1.9	3.9	5.1	14.0	*24.3*	*25.8*	*25.0*
P19	I would never visit a great city without seeing its iconic monuments	3.9	4.3	7.0	10.2	*18.9*	*23.8*	*31.9*
P20	I would never travel to a destination with high pollution levels	4.6	10.3	*18.3*	*19.8*	*15.9*	*17.4*	*13.6*
P21	For me, it's important to see exotic things or that are very different from my culture	1.5	4.9	6.7	16.5	*20.8*	*26.0*	*23.6*
P22	When I return from a vacation, I always bring souvenirs for me, family or friends	4.2	6.8	4.4	10.0	*16.5*	*22.3*	*35.7*
P23	If a destination is "in vogue" or appears in the media, I feel more like visiting it	*14.6*	*14.7*	*17.0*	*22.1*	*16.4*	9.4	5.8
P24	I would be willing to travel in a group organized by a travel agency	8.4	10.2	11.5	*15.8*	*17.6*	*18.2*	*18.3*
P25	I would never go on vacation with strangers (making common trips and meals)	*16.3*	*17.4*	*17.9*	*16.5*	11.7	10.7	9.5
P26	If I were to travel in a group, I would rather do it with people similar to me	3.0	3.9	7.3	*20.1*	*23.9*	*23.6*	*18.3*
P27	I would never travel in a group due to privacy reasons	*34.2*	*21.4*	*15.4*	14.1	6.6	4.0	4.4
P28	I would be incapable of traveling to a high criminality rate / armed conflict destination	2.9	6.9	7.6	9.7	*13.7*	*21.5*	*37.7*
P29	I like to visit uncommon places or observe peculiar things (e.g., world records, pop icons, historical items, etc.)	2.2	4.6	8.2	*17.9*	*24.9*	*23.7*	*18.5*
P30	I'm afraid of:			P31	When planning vacations, I generally prefer to go a place with:			
	Heights	*30.9*			Cold weather	2.5		
	Traveling on water	3.4			Warm weather	*27.6*		
	Flying	3.6			Hot weather	*35.0*		
	Being under water	*11.6*			I don't have a preference	*34.9*		
	Confined spaces	*14.1*						
	Other	5.4						
	I have no fears	*31.0*						
P34	Considering an itinerary / vacation plan presented by a travel agency, I would prefer:	To have a complete proposal, with everything defined, 'ready to use'		24.3	To be involved in the choice process, have more control and monitor all stages of the process			*75.7*

The highest values that contribute to most of the responses are italics, and the values that are similarly distributed are underlined

Some questions are not shown as they are related to another ongoing study

Much information can be obtained from the collected responses, but only the relevant for this study is presented. Most respondents: prefer outdoor activities but are not willing to take physical risks; like to study the destination’s history prior to traveling but consider it is not important to plan the vacation days in advance and that there should be no time schedules; want the destination to include cultural/learning components and try to include as many attractions as possible; are not worried if there is no mobile phone network available; like destinations where few people have been to, considering important to see exotic attractions or different from their culture, but would never visit an important city without seeing its iconic monuments, not feeling more keen to visit a destination for being “in vogue” or mediatized; would not travel to a highly polluted or high criminality/armed conflicts destination; consider important the accommodation’s comfort; always buy souvenirs; would accept a travel package from a travel agency but would like to be involved in the choice process; are not incommoded if they have to spend vacations with strangers or travel in a group organized by a travel agency, but would prefer to travel with tourists similar to them, all corresponding to a data distribution with positive or negative skewness. It is important to notice that although these results represent the median tourist, they do not mean that for each tourist the preferences cluster this way.

Curiously, there is a clear “balanced” division in some responses: 40% of the respondents would prefer to dine with at most 5 people and 43% would not; 41% is not afraid of getting ill or accidents while on vacations and 46% is; 33% care about the money spent on vacation and 31% do not care; 41% agree it is always better to travel in group, regardless of the destination, and 40% do not; 41% are not afraid of getting lost in a distant country, but 45% are; 32% prefer to have a good surprise while 28% prefer to fulfill expectations; 30% are indifferent if they contribute to the destination’s local economy but 34% like to feel they contribute. Regarding the destination’s weather conditions, 35% prefer hot weather, but also 35% do not have a preference, and 28% like warm weather. Cold weather is not a choice for the respondents. Most participants have some sort of phobias/fears (e.g., fear of heights, confined spaces, etc.).

Probably not all preferences and concerns were chosen by the same type of participants. Do personality dimensions predict travel-related preferences and concerns? If so, which ones? In Sect. 4.2.4, we answer those questions.

### How does personality predict preferences for tourist attractions, travel motivations, preferences and concerns?

In this section, we present the results of the EFA and CFA performed on the questionnaire items for each studied travel aspect, except for personality, where we present only the CFA results.

#### Personality

The EFA of the BFI responses confirmed the Big Five personality dimensions, aggregating the items into the expected personality dimensions. CFA confirmed the EFA results (Table 6), but some items were removed due to a regression weight < 0.45 (we considered items above this threshold as the scale consistency increased), resulting in 21 items from the 44 in the original scale, as the items used in the proposed models had to represent the sample used for the study. This probably means the sample needed to be larger to maintain all the 44 items.

**Table 6:** Confirmatory Factor Analysis of the Big Five Inventory responses, confirming the 5 personality factors extracted using EFA and their respective items, the standardized regression weights between the items and factors (all items are statistically significant at ***p < 0.001 level (2-tailed)), and each factor’s Cronbach’s Alpha reliability (α)

Factor	Item	Description	Regression weight	α
Openness	5	Is original, comes up with new ideas	0.784	0.810
	15	Is ingenious, a deep thinker	0.594	
	20	Has an active imagination	0.669	
	25	Is inventive	0.796	
	40	Likes to reflect, play with ideas	0.475	
Conscientiousness	13	Is a reliable worker	0.591	0.697
	28	Perseveres until the task is finished	0.561	
	33	Does things efficiently	0.628	
	38	Makes plans and follows through with them	0.543	
Extraversion	1	Is talkative	0.462	0.761
	11	Is full of energy	0.686	
	16	Generates a lot of enthusiasm	0.738	
	36	Is outgoing, sociable	0.579	
Agreeableness	7	Is helpful and unselfish with others	0.574	0.671
	32	Is considerate and kind to almost everyone	0.534	
	42	Likes to cooperate with others	0.689	
Neuroticism	4	Is depressed, blue	0.513	0.766
	9R	Is relaxed, handles stress well	0.733	
	24R	Is emotionally stable, not easily upset	0.640	
	34R	Remains calm in tense situations	0.681	
	39	Gets nervous easily	0.608	
			Full scale α	0.685

The full scale α is also presented at the end of the table

All the dimensions’ Cronbach’s Alpha crossed the 0.60 threshold for psychological variables (John and Benet-Martínez 2000), having an acceptable to good reliability (George and Mallery 2019). The full scale α is acceptable (*α* = 0.685), confirming the items in the scale were related to the same concepts, as expected. The model fit has a *χ*^2^/df value of 3.590 (acceptable), a CFI of 0.928, GFI of 0.945, PCFI of 0.769 and PGFI of 0.712, revealing a good goodness-of-fit, and the RMSEA = 0.050 and p(RMSEA <  = 0.05) = 0.485 a very good adjustment (Marôco 2010). The scale is therefore suitable for the study.

#### Personality vs tourist attractions preference

As a result of performing the EFA on the 68 items representing the tourist attractions, several items were eliminated^4^ according to the criteria previously referred (Sect. 3), resulting in a final scale with 50 items, and 11 factors extracted that explained 64% of the total variance. The 11 factors aggregated items measuring the same concepts, as shown in Table 7 and by their high Cronbach’s Alpha reliability values.^5^ The sampling adequacy (Kaiser–Meyer–Olkin, KMO = 0.886) is good (Pestana and Gageiro 2008), and the correlation between the variables is significative (Bartlett's Test of Sphericity Sig. = 0.000, < 0.05). The reliability for the full scale is excellent (*α* = 0.914), confirming the items in the scale are all related to the same concept, and can therefore be used as a reference. The obtained factors were then named to meaningful descriptions representing the concepts we believe they symbolized.

**Table 7:** Varimax rotated component matrix for the proposed Tourism Categories, showing the 11 factors extracted using EFA and their respective items, the estimated correlations between the items and factors, and each factor’s Cronbach’s Alpha reliability (α)

Factor	Item	Description	Estimated correlation	α
Adrenaline Activities (F1)	A46	Practice climbing or bungee jumping	0.799	0.862
	A63	Do air sports (e.g., parachute jump, skydiving, gliding)	0.774	
	A68	Ski	0.734	
	A12	Practice aquatic sports (e.g., sailing, canoeing, diving, jet skiing)	0.711	
	A29	Do motorsports (e.g., karting, motocross)	0.658	
	A37	Observe sub-aquatic environments / marine life (e.g., snorkeling, submarine)	0.521	
	A59	Participate in an escape game	0.436	
Wild Nature Activities (F2)	A41	Walk in the forest / woods	0.843	0.836
	A11	Do hiking / mountaineering	0.760	
	A42	Take a walk along the river / seacoast	0.667	
	A10	Appreciate natural landscapes	0.655	
	A47	Visit mountain areas / gorges	0.618	
	A36	Visit nature or wildlife reserves	0.546	
Party, Music & Nightlife (F3)	A43	Go to a music festival/concert	0.796	0.850
	A44	Go to a dance/ballet festival	0.790	
	A45	Go to balls (dancing)	0.743	
	A48	Go to a live music bar/place	0.677	
	A17	Go to a film festival	0.596	
	A9	Go to the disco/nightclub	0.581	
	A39	Go to a thematic parade (e.g., military, electronic music)	0.472	
Sun, Water & Sand (F4)	A65	Go to the swimming pool to relax	0.803	0.815
	A64	Go to the swimming pool to swim/dive	0.745	
	A22	Go to the beach (sunbathing/ swimming)	0.707	
	A66	Have vacation on an island	0.694	
Museums, Boat trips & Viewpoints (F5)	A34	Visit viewpoints of natural landscape	0.786	0.857
	A32	Visit museums of historical themes	0.780	
	A33	Visit museums of scientific themes (e.g., planetarium, paleontology)	0.742	
	A50	Take boat trips for the historical value of the route	0.729	
	A49	Take boat trips to know the destination's coast	0.714	
	A35	Visit viewpoints of urban landscape	0.707	
Theme & Animal Parks (F6)	A15	Go to a Zoo	0.774	0.803
	A13	Go to a theme park (e.g., Disneyland Paris)	0.670	
	A5	Visit an oceanarium	0.667	
	A27	Go to a water park	0.619	
	A25	Go to a funfair (e.g., amusements such as Ferris wheel, bumper cars, etc.)	0.563	
Cultural Heritage (F7)	A20	Visit monuments (e.g., churches, cathedrals, castles, fortresses, monasteries, palaces, etc.)	0.775	0.632
	A4	Visit the historic cities/villages of the destination	0.690	
	A38	Visit large man-made constructions (e.g., bridges, tunnels, mines)	0.539	
	A3	Watch a religious celebration	0.531	
Sports & Games (F8)	A58	Hunt / fish	0.713	0.668
	A60	Watch a bullfight	0.698	
	A55	Play at the casino	0.561	
	A56	Assist to a sporting competition (e.g., watch a football game from a club of that country)	0.499	
Gastronomy Events (F9)	A1	Go to a Gastronomy Festival (food and/or drinks)	0.798	0.735
	A40	Participate in a gastronomy tour (typical and/or gourmet dishes, wine tasting)	0.770	
	A18	Taste typical local dishes	0.693	
Health & Well-being (F10)	A14	Undergo health and wellness treatments (e.g., hydrotherapy centers, mineral water resorts)	0.724	0.801
	A28	Go to a SPA / beauty center	0.626	
Natural Phenomena (F11)	A2	Watch a natural phenomenon (e.g., volcanic eruption or northern lights)	0.686	0.639
	A6	Visit caves/caverns/volcanoes	0.626	
			Full scale α	0.914

The full scale α is also presented at the end of the table

**Fig. 3:**
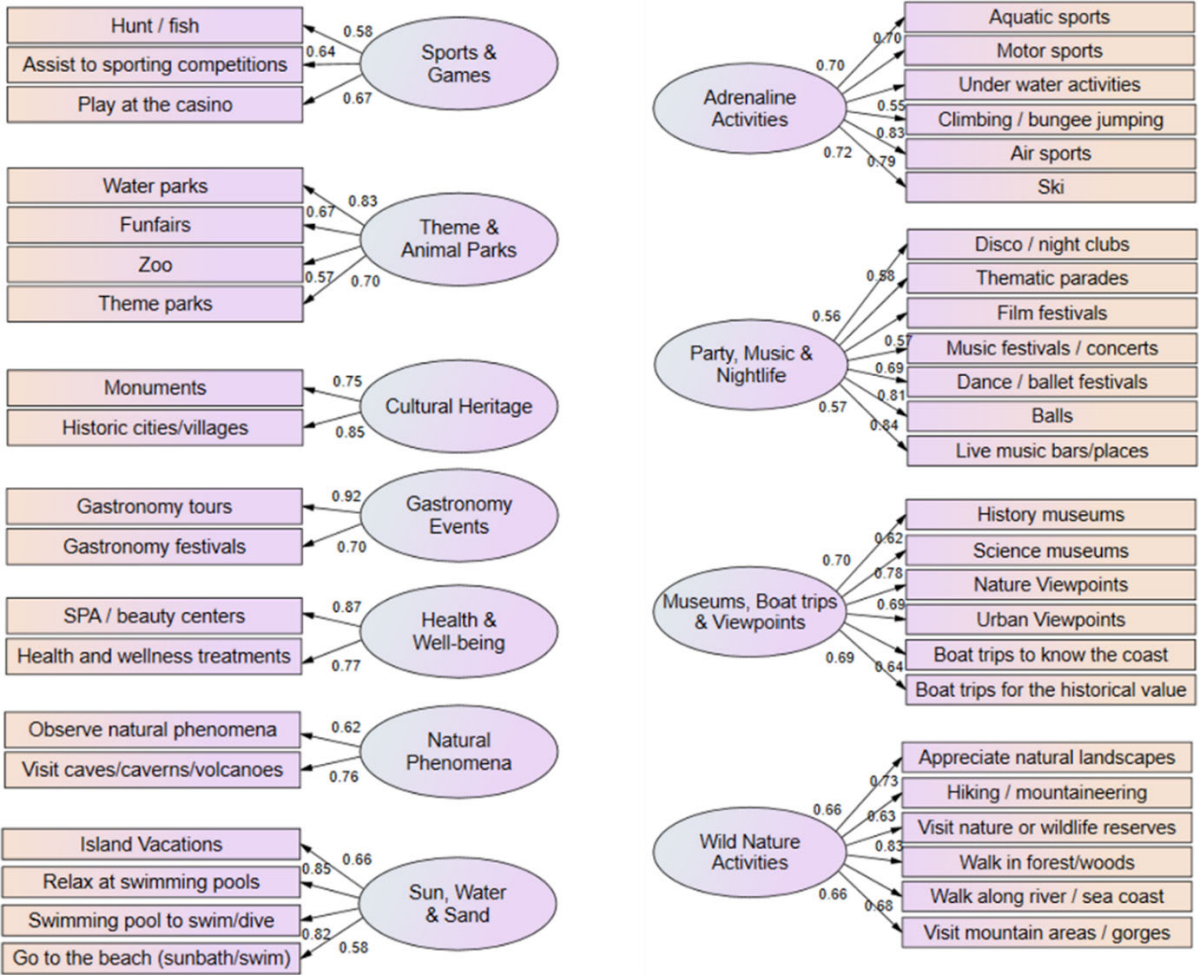
Simplified Structural Equation Model for the proposed “Tourism Categories” model, obtained using CFA. For readability, only the regression weights (standardized) are presented (*λ* ≥ 0.5)

The CFA of the extracted factors showed the observed variables (attractions items) saturated the latent variables (factors, i.e., tourism categories), confirming the items were correctly related to the proposed tourism categories (Fig. 3). The items A3, A5, A18, A38, A56, A59 and A60 had to be removed from the model, as they had a regression weight < 0.50 (see Sect. 3). All factors’ regression weights were statistically significant in the prediction of their respective items for *p* < 0.001*** (two-tailed). The model revealed an overall acceptable goodness-of-fit (*χ*^2^/df = 5.649, CFI = 0.823, GFI = 0.815, PCFI = 0.732, PGFI = 0.693, RMSEA = 0.067, p(RMSEA ≤ 0.05) = 0.000), meaning the model is valid and that the items provide an acceptable fit for the proposed model, confirming the proposed “Tourism Categories” model.

By calculating the mean value each participant scored for the Tourism Categories (factor scores), it was possible to find that there are kinds of tourist attractions that participants always like to include in their vacations, independently of their personality, as is the case of Wild Nature Activities (F2), Sun, Water & Sand (F4), Museums, Boat trips & Viewpoints (F5), Cultural heritage (F7), Gastronomy events (F9) and Natural phenomena (F11). The opposite can be said for Sports & Games (F8), where most of the respondents did not consider important to include them in their leisure vacations. This is in line with the results found in Sect. 4.1.3.^6^

**Fig. 4:**
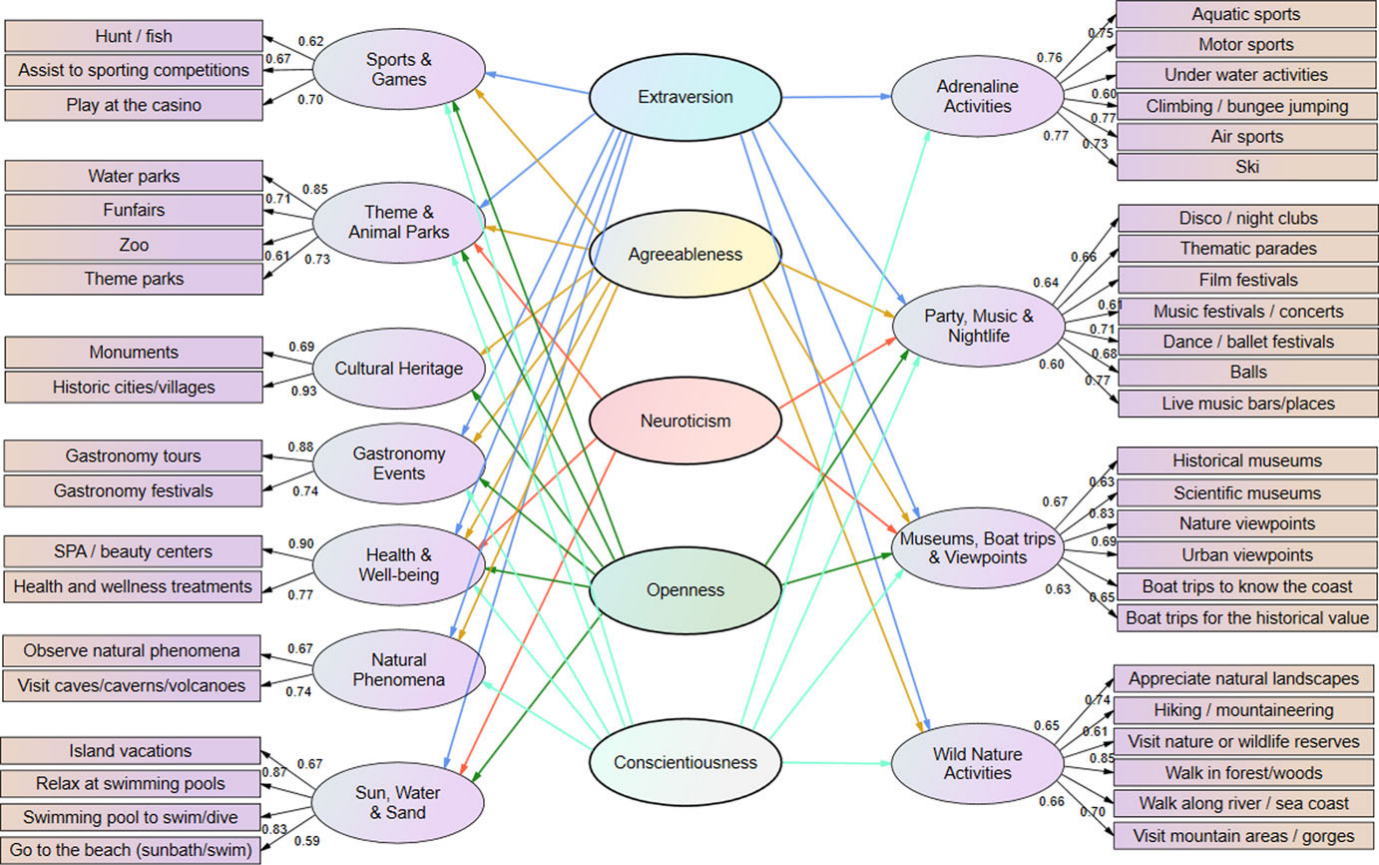
Simplified Structural Equation Model for the proposed “Personality vs Tourist Attractions Preference” model, obtained using CFA. For readability, only the regression weights (standardized) for the Tourism Categories items are presented (*λ* ≥ 0.5). The regression weights for the relationship between the personality dimensions and tourism categories are shown in Table 8

In order to answer one of this study’s research questions, the Personality dimensions were related to the Tourism Categories obtained, using CFA. As shown in Fig. 4 and Table 8, there is a clear relationship between the Big Five personality dimensions and the preference for tourist attractions.

**Table 8:** Standardized regression weights for the relationship between the BFI dimensions and the preference for tourist attractions, obtained using CFA

Tourism category	BFI dimension	Regression weight	p
Adrenaline Activities (F1)	Extraversion	***0.715***	***
	Conscientiousness	− ***0.320***	***
	Agreeableness	0.024	0.524
	Neuroticism	0.012	0.706
	Openness	− 0.039	0.224
Wild Nature Activities (F2)	Extraversion	***0.404***	***
	Agreeableness	***0.573***	***
	Conscientiousness	− ***0.223***	***
	Neuroticism	0.053	0.120
	Openness	0.017	0.608
Party, Music & Nightlife (F3)	Extraversion	***0.751***	***
	Agreeableness	− ***0.050***	***
	Neuroticism	***0.129***	***
	Openness	− ***0.115***	***
	Conscientiousness	− ***0.108***	0.003**
Sun, Water & Sand (F4)	Extraversion	***0.617***	***
	Neuroticism	***0.076***	0.026*
	Openness	− ***0.232***	***
	Agreeableness	0.008	0.827
	Conscientiousness	0.016	0.648
Museums, Boat trips & Viewpoints (F5)	Extraversion	0.063	0.097
	Agreeableness	***0.525***	***
	Neuroticism	***0.078***	0.033*
	Openness	***0.078***	0.029*
	Conscientiousness	− ***0.182***	***
Theme & Animal Parks (F6)	Extraversion	***0.790***	***
	Agreeableness	− ***0.123***	0.003**
	Neuroticism	***0.128***	***
	Openness	− ***0.204***	***
	Conscientiousness	− ***0.077***	0.026*
Cultural Heritage (F7)	Agreeableness	***0.625***	***
	Openness	− 0.006	0.085
	Extraversion	− 0.019	0.612
	Neuroticism	0.044	0.213
	Conscientiousness	− 0.006	0.864
Sports & Games (F8)	Extraversion	***0.717***	***
	Agreeableness	− ***0.309***	***
	Openness	− ***0.152***	***
	Conscientiousness	− ***0.150***	***
	Neuroticism	0.010	0.796
Gastronomy Events (F9)	Extraversion	***0.459***	***
	Agreeableness	***0.187***	***
	Openness	− ***0.116***	0.002**
	Conscientiousness	− ***0.089***	0.023*
	Neuroticism	− 0.010	0.784
Health & Well-being (F10)	Extraversion	***0.649***	***
	Agreeableness	− ***0.168***	***
	Neuroticism	***0.144***	***
	Openness	− ***0.143***	***
	Conscientiousness	***0.079***	0.029*
Natural Phenomena (F11)	Extraversion	***0.336***	***
	Agreeableness	***0.605***	***
	Conscientiousness	− ***0.365***	***
	Neuroticism	0.045	0.246
	Openness	0.020	0.598

Statistically significant values are in bold and italics (*p < 0.05 (2-tailed), **p < 0.01 (2-tailed), ***p < 0.001 (2-tailed))

The model’s fit indicators show an overall acceptable goodness-of-fit (*χ*^2^/df = 4.409, CFI = 0.771, GFI = 0.767, PCFI = 0.721, PGFI = 0.696, RMSEA = 0.057, p(RMSEA ≤ 0.05) = 0.000), suggesting the items provide a satisfactory fit, confirming the proposed “Personality vs Tourist Attractions Preference” model.

By analyzing the model, interesting relationships were found, confirming most results in our previous study (Alves et al. 2020). Individuals with a high preference for Adrenaline Activities show a high extraversion and low conscientiousness. This is in line with the findings reported in literature by Jani (2014b) and Delic et al. (2016), and with the dimensions themselves, since a positive extraversion is related to individuals who are adventurous, daring, and that seek excitement, taking unnecessary risks for adrenaline, and a negative conscientiousness to persons who are more spontaneous, less thoughtful and cautious (Costa Jr et al. 1995). The other personality dimensions did not present statistically significant values to be considered.

Wild Nature Activities are associated with the Nature category extracted in the previous study, but the item related to caves/volcanoes went to a new category representing natural phenomena, better refining the proposed model, which was accomplished by the sample improvement. Wild Nature Activities are preferred by extraverted and agreeable persons with low conscientiousness, which is in line with the dimensions’ definition, since extraverted individuals are more energetic, have a high level of activity and seek excitement and adventures, which can be accomplished by performing the wild nature activities proposed in our model. The same can be said for agreeableness, where we can easily relate traits like being generous, empathetic, unable to manipulate others, and to put the interests of others first, to concerns for nature. Regarding the low conscientiousness, if we look at type of activities that can be performed in wild nature, such as mountaineering, visit gorges, do safaris, and so on, we can find that it requires some stomach and less caution, which are characteristics of a low conscientious person. These results confirm the findings related to extraversion by Schneider and Vogt (2012), Plog (2002), Bujisic et al. (2015), Jani (2014b), Neidhardt et al. (2015), and Delic et al. (2016); to agreeableness by Hirsh (2010) and Kvasova (2015); and to conscientiousness by Jani (2014b) and Delic et al. (2016).

All five personality dimensions were found to predict preferences for Party, Music & Nightlife activities/attractions, with negative values for openness to experience, conscientiousness, and agreeableness, and positive for neuroticism with a very strong influence from extraversion. First, we can see that the duplication of respondents in this study brought a more refined model, dropping the items related to attending opera/theater and other cultural events, which were contributing to a positive agreeableness and openness to experience in the previous study where we were forced to name the tourism category with a different name. The remaining items explain the negative values of those two dimensions. Although the agreeableness weight is close to zero, and not considered relevant for the tourism category, nightlife is associated with “violent” behaviors. A negative openness may reveal not so intellectually oriented or interested in art or educative programs persons. Less thoughtful and cautious, who are willing to spend large amounts of money for momentaneous pleasure, and more spontaneous persons can explain the negative conscientiousness. The strong influence of extraversion is related to very sociable, energetic, excitement-seeking and high-spirited individuals, characteristics commonly associated with nightlife activities and events with large groups of people. To the best of our knowledge, we were the first to study this type of activities and relate them to the Big Five.

To swim or relax at the beach / swimming pool, or spend vacations on an island, are activities strongly related to high extraversion, negative openness and slightly neurotic persons (in line with the Sun Lover type Delic et al. 2016; Gibson and Yiannakis 2002)). These results support the ones found in the previous study, except openness, which now is negative, supporting the outcomes found by Jani (2014b) for that dimension, which are related to spend time with family and lay at the beach. Neuroticism can be related to the need for predictable vacations, which can be found in typical beach/hotel-related vacations.

The categories Museums & Landscapes and Boat Tours from the previous study merged into the new category Museums, Boat trips & Viewpoints, dropping the item related to boating just for the pleasure of it. The EFA in the increased sample allowed to find similarities between the two factors, proposing they should belong to the same factor. They all have in common to view/appreciate some natural or historical scenery, which was not the case of boating for pleasure. These preferences were found to be predicted by four personality dimensions, with a stronger influence from a positive agreeableness and negative conscientiousness, probably because less cautious people are more willing to take boat trips, and since viewpoints are generally located in high places, high conscientious persons may not be willing to go as they might be more susceptible to heights. The other two dimensions slightly positively influenced the preference, having a small relevance in the prediction.

Going to a water park, funfairs, zoo or theme parks are activities preferred by highly extraverted persons (energetic and excitement-seeking), slightly neurotic (individuals that feel more comfortable with friends and family), with negative agreeableness, openness (individuals who prefer familiarity, standard and not so intellectually challenging activities) and conscientiousness (revealing persons that are willing to take minor risks). With the new data, we observed a twist in the impact of agreeableness, which now is negative.

Monuments and historic cities/villages are the type of attractions preferred by highly agreeable persons, probably the ones that easily accompany family and friends just to make them happy, confirming the results found by Neidhardt et al. (2015) and Jani (2014b). Contrary to our previous study, we could not find relationship between the other four personality dimensions.

Sports & Games, like to enjoy sporting competitions, play at the casino, and hunt/fish, are preferences predicted by high extraversion values, which can derive from the energy, excitement and gregariousness inherent to this type of activities, but also to the need of competitiveness and dominating/be the best, confirming the findings of Schneider and Vogt (2012) and Neidhardt et al. (2015), but negative agreeableness, openness and conscientiousness, verifying the findings of Jani (2014b), who reported the Gamer type was related to low agreeableness and conscientiousness individuals. The difference in agreeableness from the previous study can be due to the items removed related to practicing ball sports and escape rooms, which are activities that involve cooperativeness and more open individuals. Negative openness and conscientiousness might by related to individuals who are willing to break rules and act without thinking.

Gastronomy tours/festivals are positively sought by extraverted and agreeable individuals with some negative openness and conscientiousness. This is in line with the results we previously found that who enjoys wine and food are generally high-spirited and cheerful persons. A low conscientiousness and openness can be due to less awareness or not caring for health issues persons, generally having a great pleasure in food/wine tasting and/or “addicted” in consuming more than needed allied to a low intellect, as this category is not directed for fine dining activities.

All personality dimensions are related to Health & Wellbeing (attending SPA/beauty centers and health and wellness treatments), with a clear prediction by highly extraverted (are not worried about exhibiting their body/intimacy), slightly conscientious (care for their health/wellbeing) and neurotic individuals (again worried for their health, or that stress out easily, being this sort of activities a way of relaxing), with low openness and agreeableness (more conservative, preferring routine and more interested in their own problems).

Finally, a new tourism category related to observing Natural Phenomena (like visiting caves/volcanoes or assisting to northern lights, volcanic eruptions) arose. A positive agreeableness is the most weighting dimension, followed by extraversion, and a negative conscientiousness, which is the same profile as for Wild Nature Activities.

Although some personality dimensions did not have a significant correlation to the choice of certain tourist attractions, it does not mean those correlations do not exist, but that a greater and more representative sample for each type of profile is needed. Also, it is important to note that the results found show that only some characteristics from each personality dimension explain the preferences for tourist attractions, indicating that the preferences could be finer predicted by using a questionnaire to evaluate each dimension’s six traits more precisely, supporting the issues reported by Yee et al. (2011). For example, a person considered extraverted may not be a risk taker or like adrenaline activities. It wouldn’t be very good if the RS suggested bungee jumping to the tourist.

All the different tourism categorizations and personality dimensions fall short when compared to the individual experience that a specific destination/attraction can offer, for example, the medieval fair in Sines, Portugal, is one of the greatest fairs, recreating historical moments, but there are others that are simple and are more commercially oriented.

#### Personality vs traveling motivations

As a result of performing the EFA on the 28 items representing the traveling motivations, four items were eliminated^7^ according to the criteria previously referred (Sect. 3), resulting in a final scale with 24 items, and 6 factors extracted that explained 62% of the total variance. The 6 factors aggregated items measuring the same concepts or somehow related, as shown in Table 9 and by their high Cronbach’s Alpha reliability values. The sampling adequacy (Kaiser–Meyer–Olkin, KMO = 0.853) is good (Pestana and Gageiro 2008), and the correlation between the variables is significative (Bartlett's Test of Sphericity Sig. = 0.000, < 0.05). The reliability for the full scale is good (*α* = 0.875), confirming the items in the scale are all related to traveling motivations, and can therefore be used as a reference. The obtained factors were named to meaningful descriptions representing the concepts we believe they symbolized.

**Table 9:** Varimax rotated component matrix for the proposed traveling motivations, showing the 6 factors extracted using EFA and their respective items, the estimated correlations between the items and factors, and each factor’s Cronbach’s Alpha reliability (α)

Factor	Item	Description	Estimated correlation	α
Self-development & Reliance (FM1)	M20	Working on my personal/spiritual values	0.723	0.828
	M6	Understanding more about myself	0.715	
	M18	Developing my skills and abilities	0.685	
	M19	Being near considerate people	0.628	
	M5	Being with respectful people	0.599	
	M4	Develop my personal interests	0.523	
Connectedness & Recognition (FM2)	M9	Having romantic relationships	0.717	0.784
	M10	Showing others I can do it	0.712	
	M24	Being recognized by other people	0.688	
	M23	Meeting amorous partners	0.676	
	M22	Reflecting on past memories	0.622	
	M8	Thinking about good times I’ve had in the past	0.536	
Novelty & Excitement (FM3)	M25	Experiencing something different	0.762	0.745
	M17	Experiencing thrills	0.699	
	M3	Having daring/adventuresome experiences	0.691	
	M11	Feeling the special atmosphere of the vacation destination	0.557	
Bond & Relax (FM4)	M13	Doing something with my family/friend(s)	0.834	0.713
	M27	Strengthening relationships with my family/friend(s)	0.733	
	M12	Getting away from everyday psychological stress/pressure	0.601	
Nature enjoyment (FM5)	M1	Being close to nature	0.858	0.845
	M15	Getting a better appreciation of nature	0.824	
Escape obligations (FM6)	M14	Being obligated to no one	0.756	0.599
	M26	Getting away from the usual demands of life	0.609	
	M28	Being independent	0.567	
			Full scale α	0.875

The full scale α is also presented at the end of the table

As can be seen in Table 9, except the four items that add to be removed and M12 and M26, all pairs of items that were measuring the same concepts in Pearce and Lee (2005) scale aggregated together after the EFA. We can also see that the pairs of items belonging to different dimensions in Pearce and Lee (2005) aggregated together in this study to constitute a new dimension, reducing the sample dimension, meaning the EFA considered they were measuring similar concepts, which is enforced by the obtained high reliability values, except for the Escape Obligations factor, which was on the limit of acceptance.

**Fig. 5:**
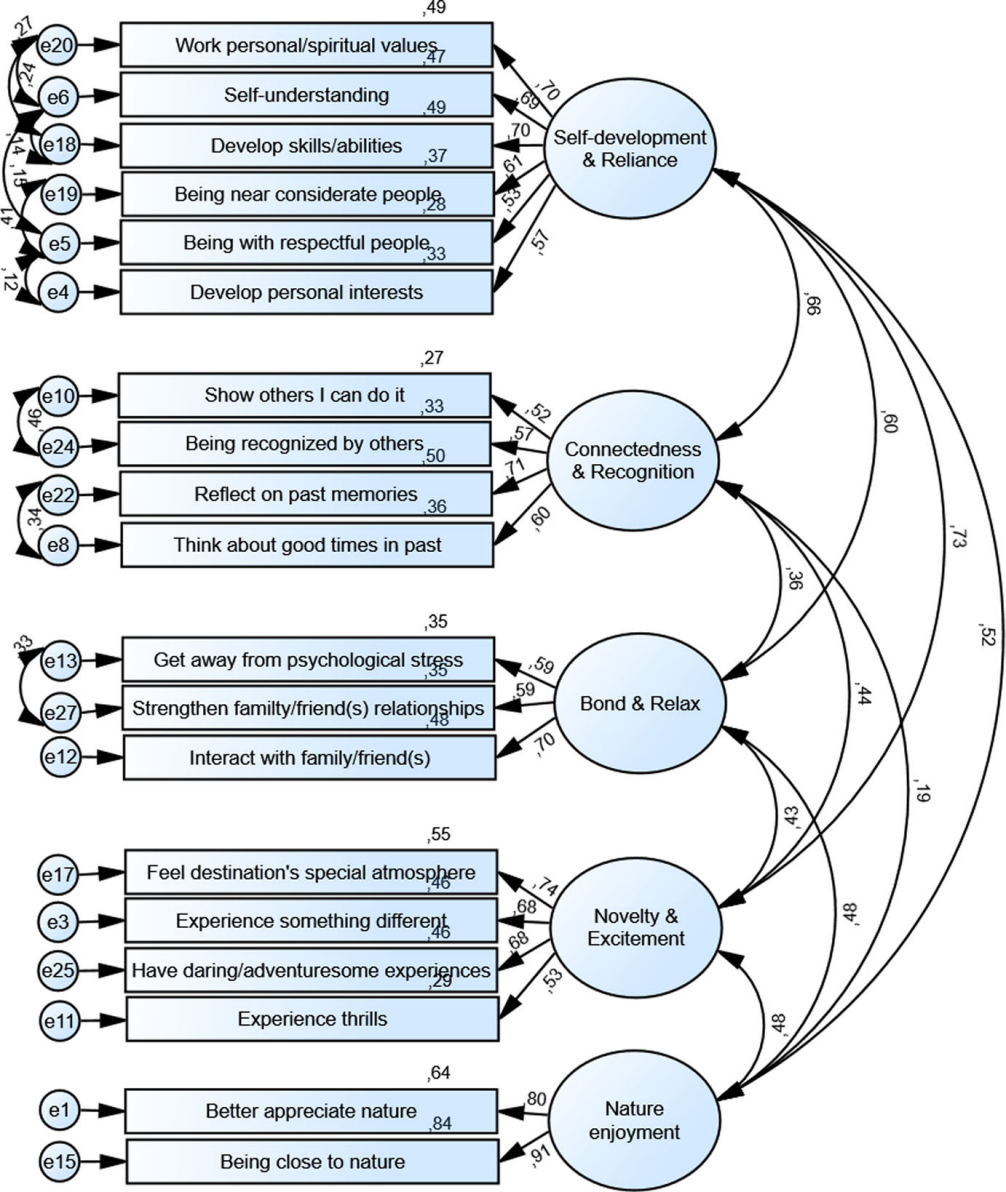
Simplified Structural Equation Model for the proposed “Traveling Motivations” model, obtained using CFA. The five factors’ regression weights were statistically significant in the prediction of their respective items for *p* < 0.001*** (two-tailed)

When performing the CFA, items M9 and M23, both related to romance, were removed from the Connectedness & Recognition factor due to a regression weight < 0.50. We also decided to remove the Escape Obligations factor, not only for having a low reliability, but because it had two items with a regression weight < 0.50, resulting in the model presented in Fig. 5. The five factors’ regression weights were statistically significant in the prediction of their respective items for *p* < 0.001*** (two-tailed). The model revealed an overall good goodness-of-fit (*χ*^2^/df = 5.999, CFI = 0.912, GFI = 0.921, PCFI = 0.709, PGFI = 0.645, RMSEA = 0.070, p(RMSEA ≤ 0.05) = 0.000), meaning the model is valid for the study and the items provide a good fit, confirming the proposed “Traveling Motivations” model. We can also observe the model includes the most common traveling motives found in literature: Novelty & Excitement (Exploration), Nature enjoyment (Nature experiences), and Bond & Relax (Relaxation/Escapism).

**Fig. 6:**
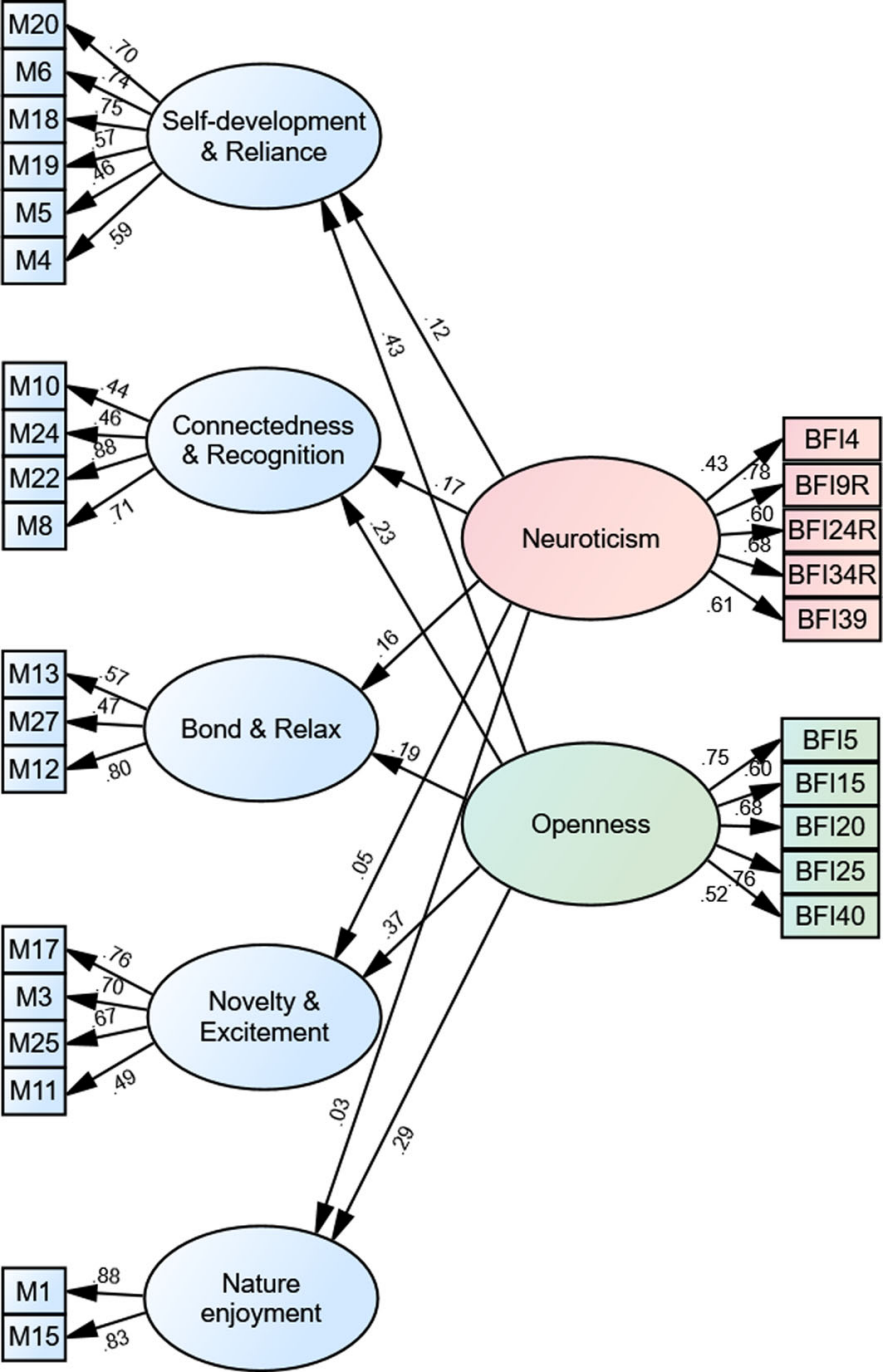
Simplified Structural Equation Model for the proposed “Personality vs Traveling Motivations” model, obtained using CFA. For readability, the error variables have been removed. The items label is shown in Tables 6 and 9

The SEM to confirm what personality dimensions were predicting which travel motivations (Fig. 6) revealed an overall acceptable fit (*χ*^2^/df = 6.379, CFI = 0.822, GFI = 0.849, PCFI = 0.721, PGFI = 0.695, RMSEA = 0.072, p(RMSEA ≤ 0.05) = 0.000), confirming the model is valid for the study and the items provide an acceptable fit, confirming the proposed “Personality vs Traveling Motivations” model.

At a first impression, it might seem difficult to explain the observed correlations. We believe the statistically significant positive influence of neuroticism on Self-development & Reliance, Connectedness & Recognition, and Novelty & Excitement motivations (Table 10) is related to individuals who are self-conscientious (internal control locus) of the need to work on those characteristics/capacities from an emotional intelligence perspective, having the capability of looking at themselves, recognizing the need to contact with others and themselves, which can be good to open horizons and have different perspectives.

We can say openness to experience is somehow related to all tourism motivations, being associated with experiencing different things, curiosity, having a greater weight on Self-development & Reliance and Bond & Relax, motivations strongly related to individuals with a higher intellect, that need to be stimulated, prone to wander the mind off, and empathetic to self and others’ feelings. The motivations related to openness are supported by Abbate and Di Nuovo (2013); Scaffidi Abbate et al. (2017) and Kashdan et al. (2009) studies.

Curiously, the agreeableness, extraversion and conscientiousness personality dimensions could not predict the proposed traveling motivations; actually they had to be removed from the model due to very low regression weights in the corresponding items. As motivations to travel depend on many factors, such as the context, destination, traveler’s mood, time of year, companions, etc., it can explain why not all dimensions could be related to the motivations for traveling (Table 10).

**Table 10:** Standardized regression weights for the relationship between the BFI dimensions and traveling motivations, obtained using CFA

Factor	BFI dimension	Regression weight	p
Self-development & Reliance (FM1)	Neuroticism	***0.125***	0.001**
	Openness	***0.429***	***
Connectedness & Recognition (FM2)	Neuroticism	***0.169***	***
	Openness	***0.226***	***
Novelty & Excitement (FM3)	Neuroticism	***0.156***	***
	Openness	***0.189***	***
Bond & Relax (FM4)	Neuroticism	0.049	0.205
	Openness	***0.372***	***
Nature enjoyment (FM5)	Neuroticism	0.028	0.450
	Openness	***0.285***	***

Statistically significant values are in bold and italics (**p < 0.01 (2-tailed), ***p < 0.001 (2-tailed))

#### Personality vs travel-related preferences and concerns

As a result of performing the EFA on the 29 items representing the relevant travel-related preferences and concerns for this study, several items were eliminated^8^ according to the criteria previously referred (Sect. 3), resulting in a final scale with 18 items, and 4 factors extracted that explained 50% of the total variance. The 4 factors aggregated items measuring the same concepts, as shown in Table 11 and by acceptable Cronbach’s Alpha reliability values.^9^ The sampling adequacy (Kaiser–Meyer–Olkin, KMO = 0.805) is good (Pestana and Gageiro 2008), and the correlation between the variables is significative (Bartlett's Test of Sphericity Sig. = 0.000, < 0.05). The reliability for the full scale is acceptable (*α* = 0.682), confirming the items in the scale are all related to the same concept, and can therefore be used as a reference. The obtained factors were then named to meaningful descriptions representing the concepts we believe they symbolized.

**Table 11:** Varimax rotated component matrix for the proposed Travel-Related Preferences and Concerns, showing the 4 factors extracted using EFA and their respective items, the estimated correlations between the items and factors, and each factor’s Cronbach’s Alpha reliability (α)

Factor	Item	Description	Estimated correlation	α
Previsibility & Safety (FP1)	P14	For me, to feel comfort is always the most important (quality of facilities / products)	0.700	0.731
	P6	I would never travel to a place where there was no mobile phone network	0.690	
	P12	In a distant country, one of my worst fears would be to get lost	0.682	
	P15	For me, to fulfill expectations is more important than a good surprise	0.564	
	P5	I am afraid of getting ill or having accidents while away on vacations	0.551	
	P2	Under no circumstances I like to take risks related to my physical integrity	0.531	
	P28	I would be incapable of traveling to a high criminality rate / armed conflict destination	0.504	
Cultural & Learning Experiences (FP2)	P18	For me, a good vacation must include a cultural / learning component	0.793	0.782
	P4	When going on vacations I take into account the destination's cultural offer	0.783	
	P10	Before traveling I like to know/study the history of the destination	0.703	
	P19	I would never visit a great city without seeing its iconic monuments	0.671	
	P7	When planning vacations, I try to include as many places/attractions as possible	0.607	
Uniqueness & Exoticness (FP3)	P21	For me, it is important to see exotic things or that are very different from my culture	0.729	0.615
	P29	I like to visit uncommon places or observe peculiar things (e.g., world records, pop icons, historical items, etc.)	0.688	
	P13	I like to go where few people have been to before	0.679	
Familiarity (FP4)	P27	I would never travel in a group due to privacy reasons	0.777	0.608
	P24R	I would be willing to travel in a group organized by a travel agency	0.772	
	P25	I would never go on vacation with strangers (making common trips and meals)	0.659	
			Full scale α	0.682

The full scale α is also presented at the end of the table. R denotes reversed questions

The EFA on the items for the travel-related preferences and concerns revealed interesting and not so obvious aggregations, showing many items were measuring the same concepts or somehow related. Regarding concerns, the participants consider important to feel safe and comfortable, are not willing to take risks regarding the physical integrity and want to have some sort of previsibility to avoid uncomfortable or risky events, preferring the familiar to the unknown, which is easier when traveling with familiars or friends, instead of strangers in a group. As for travel-related preferences, to have cultural experiences, like visiting the most famous monuments, learn about the destination’s history, see things different from their culture, and visit unusual/exotic places, are the most important for the respondents. All these outcomes support the results found in literature, previously detailed in Sect. 2.2.3.

**Fig. 7:**
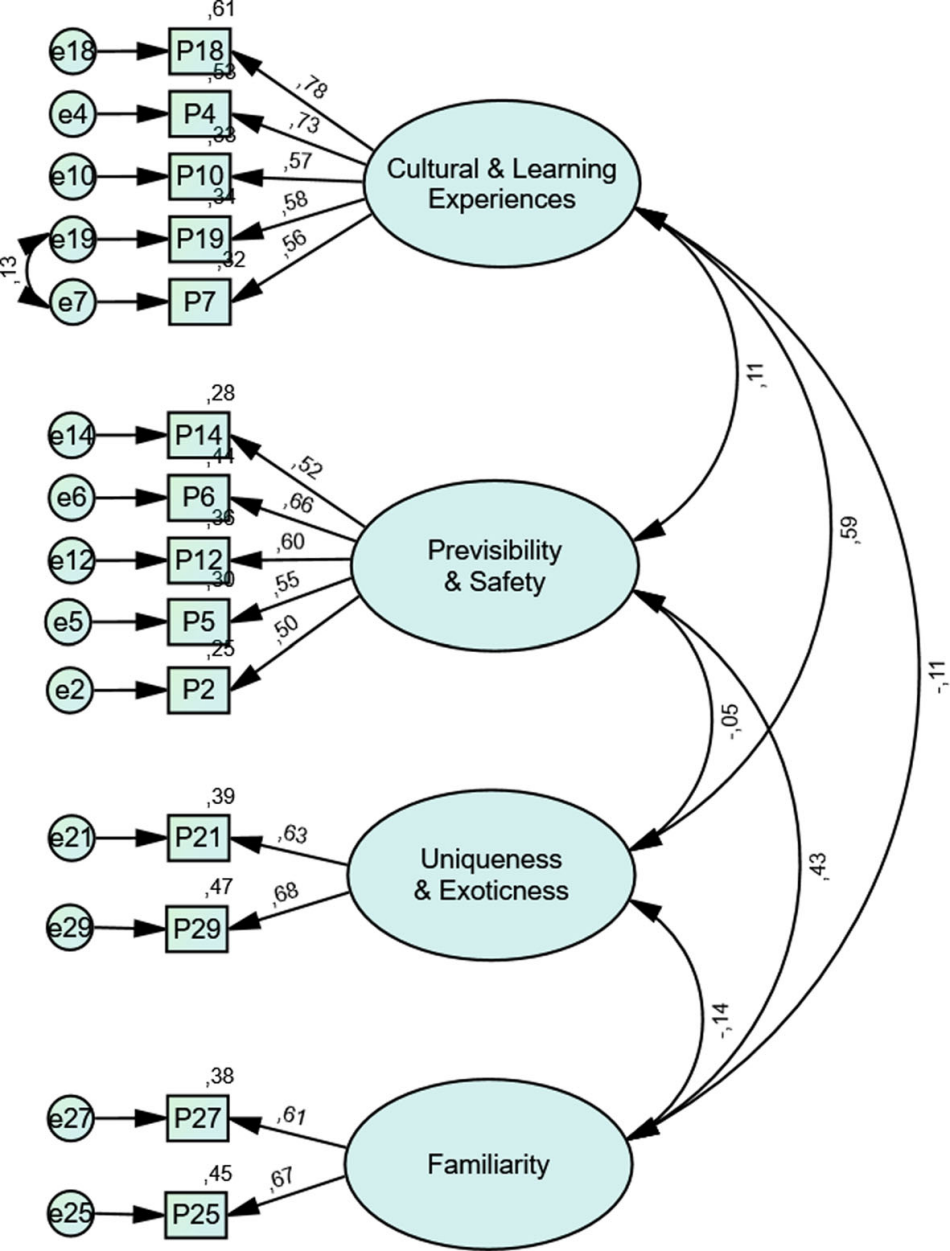
Simplified Structural Equation Model for the proposed “Travel-related Preferences and Concerns” model, obtained using CFA. The items label is shown in Table 11

The CFA on the proposed factors led to the removal of the items P13, P15, P24R, and P28, as they had a regression weight < 0.50, resulting in the model presented in Fig. 7. The four factors’ regression weights were statistically significant in the prediction of their respective items for *p* < 0.001*** (two-tailed). The model revealed an overall very good goodness-of-fit (χ^2^/df = 2.754, CFI = 0.958, GFI = 0.974, PCFI = 0.737, PGFI = 0.649, RMSEA = 0.041, p(RMSEA ≤ 0.05) = 0.982), meaning the model is valid for the study and the items provide a very good fit, confirming the proposed “Travel-related Preferences and Concerns” model.

**Fig. 8:**
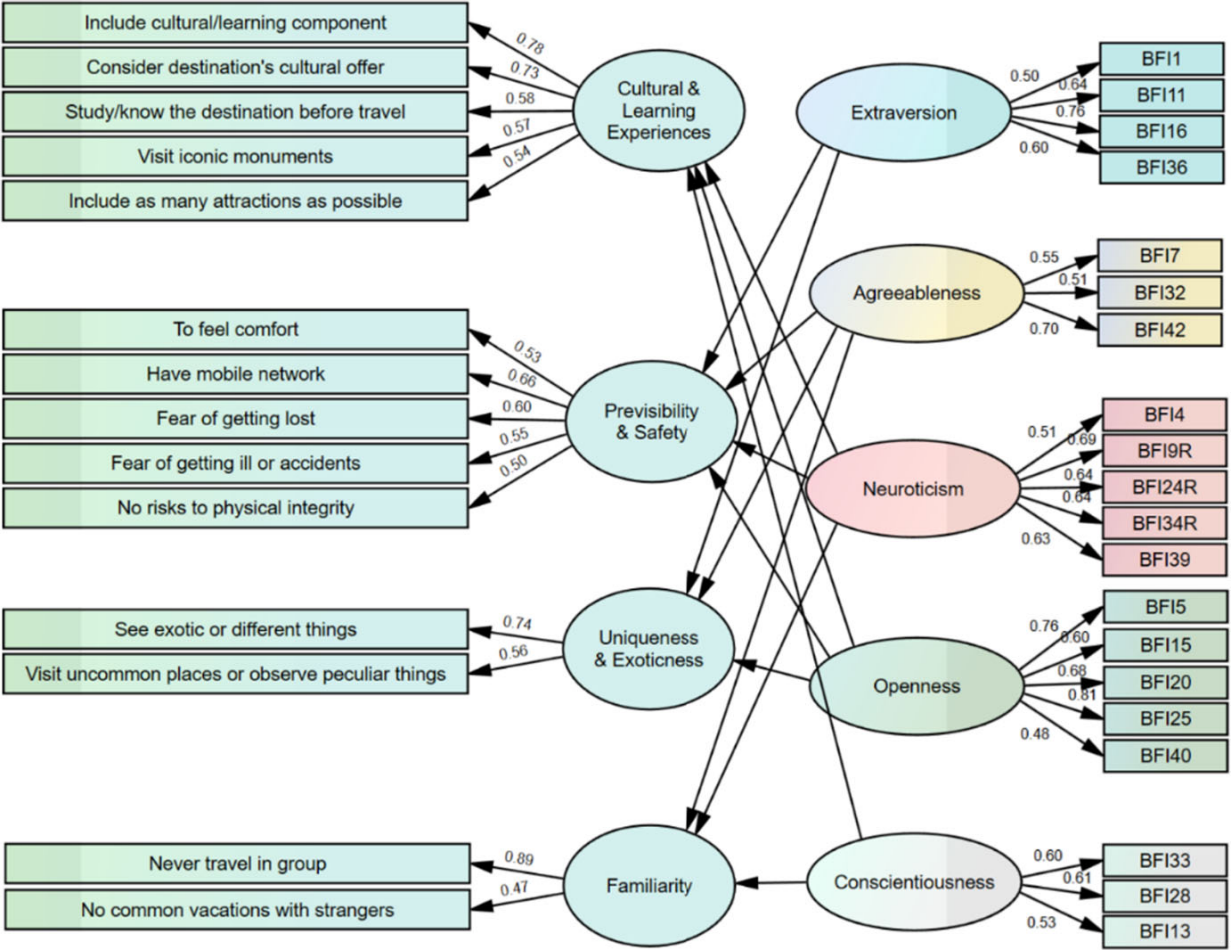
Simplified Structural Equation Model for the proposed “Personality vs Travel-related Preferences and Concerns” model, obtained using CFA. For readability, only the regression weights (standardized) for the traveling concerns and BFI items are presented (*λ* ≥ 0.5). The standardized regression weights for the relationship between the personality dimensions and traveling concerns can be found in Table 12. The BFI items label is shown in Table 6

The SEM to confirm what personality dimensions were predicting which travel-related preferences and concerns (Fig. 8) revealed an overall acceptable fit (*χ*^2^/df = 4.488, CFI = 0.809, GFI = 0.877, PCFI = 0.716, PGFI = 0.733, RMSEA = 0.058, p(RMSEA ≤ 0.05) = 0.000), confirming the model is valid for the study and the items provide an acceptable fit, confirming the proposed “Prsonality vs Travel-related Preferences and Concerns” model.

All personality dimensions are related to travel-related preferences and concerns, although with lower regression weights compared to the tourism categories and traveling motivations (Table 12). Individuals with a low extraversion and neuroticism, with some degree of agreeableness and openness, are more preoccupied with comfort, safety and health concerns (Previsibility & Safety), which can be confirmed by the findings of Maritz et al. (2013), Tan (2020), Carvalho et al. (2020), and Al-Omiri et al. (2021). The conscientiousness regression weight was not statistically significant to be considered.

The concern for traveling in groups of strangers (Familiarity) is predicted by a negative agreeableness (revealing individuals more concerned about themselves), neurotic (who are more comfortable with family/friends having difficulty in socializing with strangers) and conscientious persons (careful and less spontaneous). To the best of our knowledge, we could not find works relating the Big Five to this type of concerns. The other dimensions were not statistically significant.

Cultural & Learning Experiences are preferences positively sought by neurotic and conscientious persons, with a negative openness to experience. These predictions are easily explained, as neurotic are more anxious and therefore study the destination before traveling, and conscientious persons are more organized and inclined to plan their vacations in advance, which is in line with the negative openness.

Uniqueness & Exoticness is pursued by extraverted (seeking adventure, new things), agreeable (get along with others) and open to experience (sensible to beauty and patterns, curious, needing variety and novelty) persons.

The last two type of preferences can be corresponded to the choice of certain attractions, namely from the Museums, Boat trips & Viewpoints, Cultural Heritage, and Wild Nature Activities tourism categories, having several personality dimensions in common (Table 12).

**Table 12:** Standardized regression weights for the relationship between the BFI dimensions and travel-related preferences and concerns, obtained using CFA

Factor	BFI dimension	Regression weight	p
Previsibility & Safety (FP1)	Extraversion	***0.086***	0.033*
	Agreeableness	***0.241***	***
	Neuroticism	***0.080***	0.043*
	Openness	***0.148***	***
	Conscientiousness	0.049	0.274
Cultural & Learning Experiences (FP2)	Neuroticism	***0.296***	***
	Openness	− ***0.128***	0.001**
	Conscientiousness	***0.277***	***
	Extraversion	− 0.001	0.979
	Agreeableness	− 0.051	0.249
Uniqueness & Exoticness (FP3)	Extraversion	***0.254***	***
	Agreeableness	***0.193***	***
	Openness	***0.142***	0.002**
	Neuroticism	0.049	0.266
	Conscientiousness	− 0.041	0.412
Familiarity (FP4)	Agreeableness	− ***0.303***	***
	Neuroticism	***0.123***	0.006**
	Conscientiousness	***0.221***	0.004**
	Extraversion	0.019	0.627
	Openness	− 0.033	0.372

Statistically significant values are in bold and italics (*p < 0.05 (2-tailed), **p < 0.01 (2-tailed), *** p < 0.001 (2-tailed))

#### Personality vs fears and weather preferences

The questionnaire allowed to obtain very interesting and useful information. In this section, we correlated personality to fears and the type of weather preferred when on vacations, to explore if and how they were related.

As can be observed in Table 13, neuroticism is clearly associated with persons who have some sort of fear. The more neurotic a person is, the most susceptible to having fears is. Low values of extraversion and openness are also predictors of persons with more fears. This confirms the results found by Mellstrom et al. (1976), Faullant et al. (2011) and Al-Omiri et al. (2021). Although negative, agreeableness and conscientiousness were not statistically significant to be considered.

**Table 13:** Correlations between personality, fears and climate conditions preference at the destination

	Fears	Hot weather	Warm weather	Cold weather	No weather preference
Extraversion (E)	− 0.068*	0.143**	− 0.100**	− 0.076*	− 0.024
Agreeableness (A)	− 0.020	0.071*	− 0.047	− 0.139**	0.019
Conscientiousness (C)	− 0.007	0.122**	− 0.031	− 0.066*	− 0.071*
Neuroticism (N)	0.202**	− 0.034	0.078*	0.115**	− 0.078*
Openness (O)	− 0.061*	− 0.058	− 0.094**	0.071*	0.123**

The statistically significant values are bold and italics (** correlation is significant at the 0.01 level (2-tailed), * correlation is significant at the 0.05 level (2-tailed))

Weather conditions revealed to be correlated with personality, answering to another research question: yes, personality is an influencing factor in the preference for weather conditions and consequently, in the choice of the destination. The preference for hot weather conditions at the vacation destination is positively predicted by extraverted, agreeable and conscientious individuals. Slightly neurotic, with low extraversion and openness individuals prefer to go to destinations where the weather is warm. All personality dimensions seem to influence the choice of cold weather conditions, being sought by low extraversion, agreeableness and conscientiousness persons, but with positive neuroticism and openness values. However, it is important to notice that only 2.5% of the respondents preferred cold weather, meaning it is not a relevant choice for vacations. Open to experience individuals with low conscientiousness and neuroticism seem to not care about the weather conditions at the vacation destination.

As can be seen, all different types of weather conditions revealed different combinations of personality dimensions, with different weights, meaning they can be used to model tourists in the (G)RS to predict what destinations can be recommended in what time of the year.

We believe, to the best of our knowledge, to be the firsts to present those relationships, as no studies related to the prediction of personality in the choice of weather conditions for vacations were found.

## Automatic group recommendations just by knowing the tourists’ personality

In this study, one of the problems we propose to mitigate is the cold-start problem in (G)RS by automatically modeling the tourists profile based on their personality, namely their tourist attractions preference, traveling motivations, and travel-related preferences and concerns. The proposed models showed the referred travel aspects are related to the tourists’ personality and that the five personality dimensions have different impacts on the choice of tourist attractions, travel motivations, preferences and concerns.

To automatically model the tourists’ travel preferences, the global score^10^ for each tourist’s travel aspect, according to the analyzed sample, is calculated according to the expressions presented next. This simple mapping is intended to suppress the need for gathering large amounts of information from the users interaction to give the first recommendations, which most (G)RS depend on.

### Model 1: tourist attractions preference

Let the personality of a Tourist $T_{i}$ be represented by $P_{{T_{i} }} = \left\{ {O_{{T_{i} }} , C_{{T_{i} }} , E_{{T_{i} }} , A_{{T_{i} }} , N_{{T_{i} }} } \right\}, i \in \left\{ {1, 2, \ldots , n} \right\}$, where $O_{{T_{i} }}$ represents the Tourist’s Openness to Experience score, $C_{{T_{i} }}$ the Conscientiousness score, $E_{{T_{i} }}$ the Extraversion score, $A_{{T_{i} }}$ the Agreeableness score, and $N_{{T_{i} }}$ the Neuroticism score.

The following expressions represent the personality dimensions that significantly contribute for a tourist $T_{i}$ tourist attraction preference $AP_{{T_{i} }} \in \left[ {0, 1} \right]$, $i \in \left\{ {1, 2, \ldots , n} \right\}$, for a certain tourism category $F_{j}$, $j \in \left\{ {1, 2, \ldots , 11} \right\}$. For instance, in Eq. 1, the preference for the tourism category $F_{1}$ (Adrenaline activities) by tourist $T_{i}$ is predicted by the Extraversion regression weight for that category multiplied by the Tourist’s Extraversion score plus the Conscientiousness regression weight multiplied by the Tourist’s Conscientiousness score.

1 $$AP_{{T_{i} F_{1} }} = 0.715 \times E_{{T_{i} }} - 0.320 \times C_{{T_{i} }}$$

2 $$AP_{{T_{i} F_{2} }} = 0.404 \times E_{{T_{i} }} + 0.573 \times A_{{T_{i} }} - 0.223 \times C_{{T_{i} }}$$

3 $$AP_{{T_{i} F_{3} }} = 0.751 \times E_{{T_{i} }} - 0.050 \times A_{{T_{i} }} + 0.129 \times N_{{T_{i} }} - 0.115 \times O_{{T_{i} }} - 0.108 \times C_{{T_{i} }}$$

4 $$AP_{{T_{i} F_{4} }} = 0.617 \times E_{{T_{i} }} + 0.076 \times N_{{T_{i} }} - 0.232 \times O_{{T_{i} }}$$

5 $$AP_{{T_{i} F_{5} }} = 0.525 \times A_{{T_{i} }} + 0.078 \times N_{{T_{i} }} + 0.078 \times O_{{T_{i} }} - 0.182 \times C_{{T_{i} }}$$

6 $$AP_{{T_{i} F_{6} }} = 0.790 \times E_{{T_{i} }} - 0.123 \times A_{{T_{i} }} + 0.128 \times N_{{T_{i} }} - 0.204 \times O_{{T_{i} }} - 0.077 \times C_{{T_{i} }}$$

7 $$AP_{{T_{i} F_{7} }} = 0.625 \times A_{{T_{i} }}$$

8 $$AP_{{T_{i} F_{8} }} = 0.717 \times E_{{T_{i} }} - 0.309 \times A_{{T_{i} }} - 0.152 \times O_{{T_{i} }} - 0.150 \times C_{{T_{i} }}$$

9 $$AP_{{T_{i} F_{9} }} = 0.459 \times E_{{T_{i} }} + 0.187 \times A_{{T_{i} }} - 0.116 \times O_{{T_{i} }} - 0.089 \times C_{{T_{i} }}$$

10 $$AP_{{T_{i} F_{10} }} = 0.649 \times E_{{T_{i} }} - 0.168 \times A_{{T_{i} }} + 0.144 \times N_{{T_{i} }} - 0.143 \times O_{{T_{i} }} + 0.079 \times C_{{T_{i} }}$$

11 $$AP_{{T_{i} F_{11} }} = 0.336 \times E_{{T_{i} }} + 0.605 \times A_{{T_{i} }} - 0.365 \times C_{{T_{i} }}$$

### Model 2: traveling motivations

The tourist $T_{i}$ predicted traveling motivation $TM_{{T_{i} }} \in \left[ {0, 1} \right]$, $i \in \left\{ {1, 2, \ldots , n} \right\}$, for a certain motivation factor $FM_{j}$, $j \in \left\{ {1, 2, \ldots , 5} \right\}$ is calculated as follows:

12 $$TM_{{T_{i} FM_{1} }} = 0.125 \times N_{{T_{i} }} + 0.429 \times O_{{T_{i} }}$$

13 $$TM_{{T_{i} FM_{2} }} = 0.169 \times N_{{T_{i} }} + 0.226 \times O_{{T_{i} }}$$

14 $$TM_{{T_{i} FM_{3} }} = 0.156 \times N_{{T_{i} }} + 0.189 \times O_{{T_{i} }}$$

15 $$TM_{{T_{i} FM_{4} }} = 0.372 \times O_{{T_{i} }}$$

16 $$TM_{{T_{i} FM_{5} }} = 0.285 \times O_{{T_{i} }}$$

### Model 3: travel-related preferences and concerns

The tourist $T_{i}$ predicted travel-related preference $TP_{{T_{i} }} \in \left[ {0, 1} \right]$, $i \in \left\{ {1, 2, \ldots , n} \right\}$, for a certain travel-related preference factor $FP_{j}$, $j \in \left\{ {2, 3} \right\}$ is calculated as follows:

17 $$TP_{{T_{i} FP_{2} }} = 0.296 \times N_{{T_{i} }} - 0.128 \times O_{{T_{i} }} + 0.277 \times C_{{T_{i} }}$$

18 $$TP_{{T_{i} FP_{3} }} = 0.254 \times E_{{T_{i} }} + 0.193 \times A_{{T_{i} }} + 0.142 \times O_{{T_{i} }}$$

The tourist $T_{i}$ predicted travel-related concern $TC_{{T_{i} }} \in \left[ {0, 1} \right]$, $i \in \left\{ {1, 2, \ldots , n} \right\}$, for a certain travel-related concern factor $FP_{j}$, $j \in \left\{ {1, 4} \right\}$ is calculated as follows:

19 $$TC_{{T_{i} FP_{1} }} = 0.086 \times E_{{T_{i} }} + 0.241 \times A_{{T_{i} }} + 0.080 \times N_{{T_{i} }} + 0.148 \times O_{{T_{i} }}$$

20 $$TC_{{T_{i} FP_{4} }} = - 0.303 \times A_{{T_{i} }} + 0.123 \times N_{{T_{i} }} + 0.221 \times C_{{T_{i} }}$$

### Model 4: creating subgroups with similar interests

The other problem we propose to mitigate is the conflicting preferences in groups of tourists. As the probability of heterogeneity is greater with larger groups of tourists, if we use the proposed models to create subgroups of tourists with similar interests, we can minimize the group’s heterogeneity and conflicts of interest, which in turn facilitates reaching a consensus in the recommendation process and the generation of more precise recommendations to the subgroups. This type of aggregation can be advantageous in promoting more socialization and the creation of bounds between the group members (Alves et al. 2019), especially in occasional groups of tourists (e.g., promoted by travel agencies, companies, or other organized groups).

Considering it only makes sense to have (sub)groups of 3 or more elements, let $G_{e} = \left\{ {T_{i} , \ldots , T_{n} } \right\}$ be a group of Tourists for a certain excursion $e$, where $e, i \in \left\{ {1, 2, \ldots , n} \right\}$ and $G_{e} \ge 3$; $AP_{{T_{i} }} = \left\{ {AP_{{T_{i} F_{1} }} , \ldots , AP_{{T_{i} F_{11} }} } \right\}$ the set of tourist attractions preference of Tourist $T_{i}$, $TM_{{T_{i} }} = \left\{ {TM_{{T_{i} FM_{1} }} , \ldots , TM_{{T_{i} FM_{5} }} } \right\}$ the set of traveling motivations of Tourist $T_{i}$, $TP_{{T_{i} }} = \left\{ {TP_{{T_{i} FP_{2} }} , TP_{{T_{i} FP_{3} }} } \right\}$ the set of travel-related preferences of Tourist $T_{i}$, and $TC_{{T_{i} }} = \left\{ {TC_{{T_{i} FP_{1} }} , TC_{{T_{i} FP_{4} }} } \right\}$ the set of travel-related concerns of Tourist $T_{i}$. The Tourist’s simplified profile can be represented by $profile_{{T_{i} }} = \left\{ {AP_{{T_{i} }} , TM_{{T_{i} }} , TP_{{T_{i} }} , TC_{{T_{i} }} } \right\}$.

Let $poi_{k}$, k $\in \left\{ {1, 2, \ldots , n} \right\}$, be a point of interest representing a certain tourist attraction. Considering a point of interest has a certain personality, and therefore, according to the proposed models, it scores a certain value on each tourism category, travel motivation, travel-related preference and travel-related concern, we can represent $poi_{k}$ using the same Tourist’s profile parameters: $profile_{{poi_{k} }} = \left\{ {AP_{{poi_{k} }} , TM_{{poi_{k} }} , TP_{{poi_{k} }} , TC_{{poi_{k} }} } \right\}$.

To find out how much a certain $poi_{k}$ in the excursion destination is similar to a certain tourist $T_{i}$, we calculate the Euclidean distance between them:

21 $$d\left( {T_{i} ,poi_{k} } \right) = \sqrt {\begin{array}{*{20}l} {\left( {AP_{{T_{i} F_{1} }} - AP_{{poi_{k} F_{1} }} } \right)^{2} + \left( {AP_{{T_{i} F_{2} }} - AP_{{poi_{k} F_{2} }} } \right)^{2} + \cdots + \left( {AP_{{T_{i} F_{11} }} - AP_{{poi_{k} F_{11} }} } \right)^{2} } \hfill \\ { + \left( {TM_{{T_{i} FM_{1} }} - TM_{{poi_{k} FM_{1} }} } \right)^{2} + \cdots + \left( {TM_{{T_{i} FM_{5} }} - TM_{{poi_{k} FM_{5} }} } \right)^{2} + \left( {TP_{{T_{i} FP_{2} }} - TP_{{poi_{k} FP_{2} }} } \right)^{2} } \hfill \\ { + \left( {TP_{{T_{i} FP_{3} }} - TP_{{poi_{k} FP_{3} }} } \right)^{2} + \left( {TC_{{T_{i} FP_{1} }} - TC_{{poi_{k} FP_{1} }} } \right)^{2} + \left( {TC_{{T_{i} FP_{4} }} - TC_{{poi_{k} FP_{4} }} } \right)^{2} } \hfill \\ \end{array} }$$

Following the same logic in a previous study (Carneiro et al. 2020), we propose the algorithms presented next to create the subgroups, where $difScoresT_{i} T_{j}$ represents the difference between the maximum and minimum scores $T_{i}$ gives to the list of N preferred POI of the other tourist $T_{j}$ (see Eq. 22). The resulting $difScoresT_{i} T_{j}$ can be classified into one of five possible weight levels (Table 14).

22 $$\begin{aligned} difScoresT_{i} T_{j} & = {\text{max}}\left( {T_{i} \left( {poiScoreSum} \right)} \right) - {\text{min}}(T_{i} \left( {poiScoreSum)} \right), \\ & \quad if\max \left( {T_{i} \left( {poiScoreSum} \right)} \right) \\ & \ne {\text{min}}(T_{i} \left( {poiScoreSum)} \right) else {\text{max}}(T_{i} \left( {poiScoreSum} \right)) \\ \end{aligned}$$

**Table 14:** ${\text{difScoresT}}_{{\text{i}}} {\text{T}}_{{\text{j}}}$ weight levels

Weight level	${\text{difScoresT}}_{{\text{i}}} {\text{T}}_{{\text{j}}}$
5	$difScoresT_{i} T_{j} \ge 0.80$
4	$0.60 \le difScoresT_{i} T_{j} < 0.80$
3	$0.40 \le difScoresT_{i} T_{j} < 0.60$
2	$0.20 \le difScoresT_{i} T_{j} < 0.40$
1	$difScoresT_{i} T_{j} < 0.20$


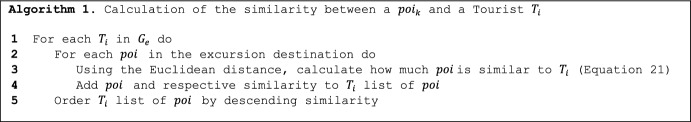



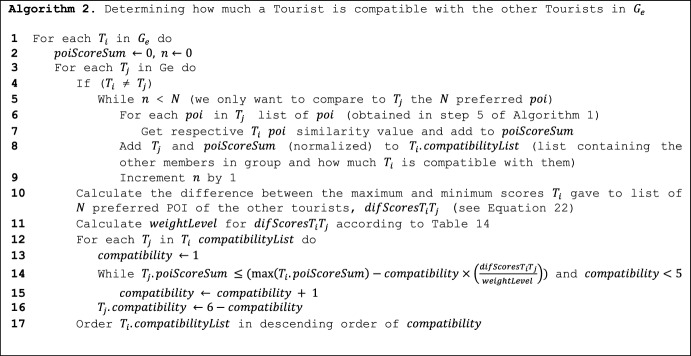



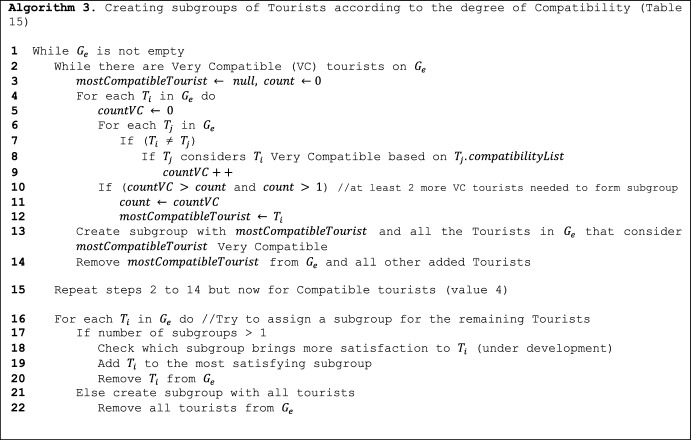


After applying Algorithm 3, the created subgroups already have the best POI recommendations (Table 15).

**Table 15:** Possible degrees of compatibility between tourists

Compatibility degree	Definition
5	Very compatible (VC)
4	Compatible (C)
3	Medium compatible (MC)
2	Few compatible (FC)
1	Least compatible (LC)

The proposed algorithms are currently being tested for accuracy, and we are developing a model to determine how many POI should be recommended according to the weight the Tourist gives to each travel aspect. For example, if the Tourist considers "Sun, Water & Sand” as the preferred category, more POI from that category should be recommended opposed to the other categories.

## Reflections and future work

The travel and tourism domain is very vast and complex, and is profoundly related to the tourists’ psychological aspects. The evolution of internet and mobile devices led to more demanding tourist consumers, eager to obtain better and emotional experiences, and consequently to the proliferation of recommender systems (RS) for tourism, aiming to provide personalized suggestions of places and attractions to visit. Personality computing came to leverage those systems by taking advantage of the psychology of tourism, leading to the creation of personality-aware RS, as it is evidenced that personality is related to the users’ preferences and solves the cold-start problem inherent to the classic RS.

Many RS for tourism can be found in literature, but few use the raw dimensions of personality to predict the tourists’ preferences, especially if we talk of GRS. We believe GRS can be greatly improved by using raw psychological aspects, such as personality, to form groups of tourists with similar preferences, providing more personalized recommendations and solving conflicting preferences. Another advantage is that this logic can be extrapolated to other domains. Although several studies on the relationship between personality and travel aspects exist, most of them are based on tourist typologies/roles, which do not disclose the kind of personality traits the tourists have, and therefore, it is hard to predict how they influence those preferences and behaviors, and consequently, are not so accurate, as not all attractions in a typology are suitable for the same tourist and a combination of typologies might be needed. In this work, we proposed to solve those limitations by finding the relationships between the Big Five personality dimensions, and the preference for a wide range of tourism categories, traveling motives and travel-related preferences and concerns, aiming to provide a solid ground for tourism GRS researchers to automatically model more accurate tourist’s profiles and create groups with similar preferences, providing more accurate and pleasing recommendations, and consequently increase the tourists’ experience and satisfaction.

Regarding the sample, this time we gathered the double of responses, with participants from more heterogenous ages and areas of formation, representing a “young” market (adults and young adults, ≤ 55 years old). However, most respondents are females (74%), meaning there can be a bias toward female preferences. The sample represents the Portuguese culture, but it can be concluded that they have many similarities with the results found in literature for other cultures. The same social desirability bias from our previous work was observed in the BFI responses to the items of the same personality dimensions (agreeableness, neuroticism, and conscientiousness), confirming the typical phenomenon of self-reporting questionnaires, although the respondents had more diverse profiles.

The “Tourism Categories” and “Personality vs Tourist Attraction Preferences” models improved their goodness-of-fit, but not as much as expected (more responses for each type of personality and gender would be needed to considerably improve the models’ fit), and certain tourism categories were finer grained as we managed to obtain a more heterogenous sample, successfully predicting the tourists' preferences based on their personality.

Relevant relationships between personality and traveling motivations were found, especially regarding high values of openness to experience, meaning personality can be a differentiating factor in predicting motivations, although needing more profound research, as motivations are highly dependent on the type of destination and context, which could be confirmed by relating motivations to tourism categories, which was not the scope of this study. Also, the “Personality vs Traveling Motivations” model needs to be improved, so all personality dimensions can be considered.

The proposed travel-related preferences aggregated into two factors that could be easily corresponded to some tourism categories. On the one hand, it was a good outcome, as it could confirm the relationship found between personality and the corresponding tourism categories; on the other hand, it might mean the items used needed to be improved to better capture other type of travel-related preferences. The travel-related concerns also aggregated into two factors, representing the participants’ fears and preoccupations, successfully predicted by their personality.

Although being predicted by personality, the proposed models need to be better tested, to determine if the personality dimensions are enough to make accurate predictions, or if other characteristics of the tourists need to be considered for better recommendations, being an ongoing study, where we are developing a mobile GRS prototype for tourism to test the models in real use-case scenarios (Alves et al. 2022). For example, during the study, we noticed that younger participants were more prone to adrenaline activities than older ones, and the results clearly showed it would be possible to obtain finer grained predictions, i.e., if we had used a personality questionnaire to obtain the 30 personality traits, instead of the 5 personality dimensions, and correlate them to the proposed travel aspects, we could have obtained more accurate predictions. This finding is supported in literature by Yee et al. (2011) and Aschwanden et al. (2021). For instance, we cannot recommend a crowded medieval fair to a high extraversion scorer who is low on the gregariousness trait. This means not only the personality information available needs to be more granular, but the context and particularities related to the attractions need to be known in advance, which we are addressing by developing an ontology of tourist attractions that will be used in the algorithm for calculating the tourist’s preference for each attraction, including the tourist’s four travel aspects preference.

With this work, we managed to provide objective relationships between the Big Five personality dimensions, and some respective traits, and the preference for a wide range of tourism categories, traveling motives and travel-related preferences and concerns, contributing with a formalization that can be used by researchers of (G)RS for tourism to automatically model the tourists’ profile based on their raw personality, being, to the best of our knowledge, the firsts to do so, mitigating the cold-start problem. Especially in the case of occasional groups (promoted or not by a tourism agency), the tourists’ profile can then be used to match the group members and create subgroups of tourists with similar interests, minimizing the groups’ heterogeneity and conflicts of interest.

To solve the problem related to filling tedious and long questionnaires on personality, we are also implementing gamification components in the GRS prototype as a proof of concept to test if we can implicitly acquire the tourists’ personality, which can then be adapted to any kind of system.

## Data Availability

The questionnaire used and the aggregated results are available at Appendix A in the following url: http://www.gecad.isep.ipp.pt/grouplanner/dissemination.html.

## Footnotes

1 This work refers only to leisure tourism.

2 Please consult http://www.gecad.isep.ipp.pt/grouplanner/dissemination.html.. The questionnaire was anonymous and confidential, and approved by the Polytechnic of Porto Data Protection Office.

3 The statistics for the BFI responses can be found at Appendix B at http://www.gecad.isep.ipp.pt/grouplanner/dissemination.html

4 Although aggregating in factors that seemed to measure the same concepts, the items with coefficients < 0.40, communalities < 0.50, that belonged to factors with a low reliability (α < 0.60), that were scoring alone, or that were scoring in more than one factor, with a difference less than 0.10, were eliminated from the sample: A7, A8, A16, A19, A21, A23, A24, A26, A30, A31, A51, A52, A53, A54, A57, A61, A62, A67.

5 Being an exploratory analysis, it is acceptable to consider factors with Cronbach’s Alpha values ≥ 0.60 (Hair et al. 2009).

6 The corresponding graphics can be found at Appendix C at http://www.gecad.isep.ipp.pt/grouplanner/dissemination.html.

7 Although aggregating in factors that seemed to measure the same concepts, the items with coefficients < 0.40, that were scoring alone, or that were scoring in more than one factor, with a difference less than 0.10, were eliminated from the sample: M2, M7, M16, M21. More details can be found at http://www.gecad.isep.ipp.pt/grouplanner/dissemination.html.

8 Although aggregating in factors that seemed to measure the same concepts, the items with coefficients < 0.40, communalities < 0.50, that belonged to factors with a low reliability (α < 0.60), that were scoring alone, or that were scoring in more than one factor, with a difference less than 0.10, were eliminated from the sample: P1, P3, P8, P9, P11, P16, P17, P20, P22, P23, P26. Please consult http://www.gecad.isep.ipp.pt/grouplanner/dissemination.html for further details.

9 Being an exploratory analysis, it is acceptable to consider factors with Cronbach’s Alpha values ≥ 0.60 (Hair et al. 2009).

10 Based on the factor score weights calculated by Amos (regression weights for predicting the unobserved variables from the observed variables).
